# Eye Tracking as a Treatment Monitoring Tool for Autism: A Multilevel Meta‐Analysis

**DOI:** 10.1002/aur.70141

**Published:** 2025-11-14

**Authors:** Christy D. Yoon, Yan Xia, Adriana Kaori Terol, Hedda Meadan, Frederick Shic

**Affiliations:** ^1^ Waisman Center, University of Wisconsin‐Madison Madison Wisconsin USA; ^2^ Department of Educational Psychology University of Illinois at Urbana‐Champaign Champaign Illinois USA; ^3^ Department of Psychiatry and Behavioral Sciences University of California, Davis, MIND Institute Sacramento California USA; ^4^ Department of Special Education and Child Development University of North Carolina at Charlotte Charlotte North Carolina USA; ^5^ Center for Child Health, Behavior and Development Seattle Children's Research Institute Seattle Washington USA; ^6^ Department of Pediatrics University of Washington School of Medicine Seattle Washington USA

**Keywords:** autism, eye tracking, intervention, meta‐analysis, treatment

## Abstract

There is a growing body of evidence suggesting a concurrent association between attentional indices measured via eye tracking and autism symptoms. This meta‐analysis examined the utility of eye tracking within longitudinal frameworks for autism interventions, including treatment monitoring and prediction of treatment response. We conducted a multivariate random‐effects meta‐analysis with a multilevel structure on 25 studies (828 autistic participants; *M*_age_ = 3–28 years) to estimate: (a) changes in eye‐tracking outcomes from pre‐ to post‐treatment (*k* = 179); and (b) the correlation between baseline eye‐tracking profiles and changes in developmental outcomes following treatment (*k* = 39). Our analysis revealed a moderate and significant summary effect size for changes in eye‐tracking outcomes from pre‐ to post‐treatment (Hedge's *g* = 0.32, *p* = 0.010). Additionally, a moderate but non‐significant summary effect size was revealed for the correlation between baseline eye‐tracking outcomes and changes in developmental outcomes following treatment (Fisher's *z* = 0.20, *p* = 0.115), with moderation effects observed based on developmental domain and sex. These findings highlight the potential of eye tracking as a tool for monitoring treatment‐induced changes in autistic individuals, while its predictive utility remains less supported. Limitations and implications are discussed.

**Summary**

- This meta‐analysis examined how eye‐tracking measures can help understand the effects of autism treatments.
- We observed changes in eye‐tracking outcomes following treatment in autistic individuals.
- However, the association between initial eye‐tracking profiles and changes in developmental outcomes following treatment was not statistically significant.
- Overall, our findings suggest that eye tracking may be useful for monitoring treatment‐related changes in autistic individuals, although its ability to predict long‐term developmental outcomes in response to treatment remains uncertain.

Autism is a complex neurodevelopmental condition characterized by a range of challenges in social communication, social interaction, and repetitive and restricted behaviors (American Psychiatric Association 2013). These defining characteristics manifest uniquely among individuals and evolve throughout their lives (Chen et al. 2024; McGovern and Sigman 2005; Waizbard‐Bartov and Miller 2023), reflecting high behavioral and clinical heterogeneity within the autism spectrum. This heterogeneity extends across differences in cognitive abilities (Ben‐Itzchak et al. 2014; Farley et al. 2009; Lage et al. 2024), sensory processing (Kadlaskar et al. 2023; Tillmann et al. 2020; Uljarević et al. 2016), language development (Pickles et al. 2014; Tager‐Flusberg 2006; Tek et al. 2014), and behavioral patterns (Faja and Nelson Darling 2019; Lord et al. 2015; Rodriguez and Thompson 2015). Consequently, a nuanced understanding of its developmental pathways is essential, necessitating tailored assessment and intervention approaches supported by sophisticated tools capable of accurately tracking developmental trajectories and predicting outcomes.

The urgency of these initiatives is amplified by the rising prevalence of autism, with recent epidemiological data estimating that approximately one in 31 children aged 8 years (Shaw et al. 2025) and one in 45 adults aged 18 to 84 years (Dietz et al. 2020) in the United States receive an autism diagnosis. This trend highlights the critical need for innovative, evidence‐based approaches for accurate diagnosis, tailored intervention, and precise treatment monitoring. Current interventions—including behavioral therapies (e.g., Pivotal Response Training; Koegel et al. 2010), pharmacological treatments (e.g., oxytocin administration; Watanabe et al. 2015), and technological interventions (e.g., virtual reality‐based social skills training; Amat et al. 2021)—aim to improve the quality of life for autistic individuals. While behavioral interventions primarily target core symptoms, existing pharmacological treatments generally address co‐occurring conditions such as anxiety, attention‐deficit/hyperactivity disorder, and irritability. Technological interventions have gained traction in recent years, offering innovative and individualized approaches for skill development and environmental support. Ongoing research explores novel pharmacological and technological interventions aimed at addressing core symptoms, although effective treatments in these domains remain to be established. Despite considerable progress, the heterogeneity inherent to autism and individual variability in treatment response (Vivanti et al. 2014) continue to pose challenges for the development, validation, and application of reliable outcome measures (Grzadzinski et al. 2020; McConachie et al. 2015).

Existing assessment tools for autism—such as the Autism Diagnostic Observation Schedule (ADOS; Lord et al. 2012) and Autism Diagnostic Interview‐Revised (ADI‐R; Lord et al. 1994) for reliable diagnoses, the Mullen Scales of Early Learning (MSEL; Mullen 1995) for developmental maturity, the Social Responsiveness Scale (SRS; Constantino 2012) for autism symptoms, and the Vineland Adaptive Behavior Scales (VABS; Sparrow et al. 2005) for adaptive functioning—remain indispensable. Nonetheless, these tools often confront limitations in measuring treatment outcomes (Bishop et al. 2019; Matson 2007). They may lack the sensitivity required to detect subtle or incremental changes (Bishop et al. 2019), particularly in social communication skills, which are often the primary targets of autism interventions (Anagnostou et al. 2015; Cunningham 2012; Yoder et al. 2013). For example, short‐term improvements in social engagement—often assessed in intervention trials lasting less than 6 months—may not be adequately captured by tools designed to evaluate long‐term developmental milestones (Grzadzinski et al. 2020; Rogers et al. 2012; Thurm et al. 2015). Additionally, these assessments often rely on subjective reports from caregivers, educators, or clinicians, potentially introducing bias (Donohue et al. 2019; Grzadzinski et al. 2020; Miller et al. 2017). This highlights the pressing need for objective, quantifiable measures that complement traditional assessments, thereby enhancing the capacity to monitor and evaluate intervention outcomes with greater precision.

Eye‐tracking technology has emerged as a promising neurobehavioral biomarker for autism (Shic 2016), providing precise, objective, and reproducible data on attentional patterns. These patterns, such as gaze directed toward faces, eyes, or social scenes, are deeply linked to the underlying mechanisms of core diagnostic features, particularly within the social communication domain (Chawarska et al. 2012; Klin et al. 2002; Major et al. 2022; Murias et al. 2018; Shic et al. 2020, 2022, 2023; Wall et al. 2023). Consequently, eye‐tracking measures have demonstrated significant associations with widely used clinical assessments for autism (Jones et al. 2023). Prior systematic reviews and meta‐analyses' findings further support the significance of these measures (Riddiford et al. 2022; Yoon et al. 2024, 2025). For example, a comprehensive meta‐analysis of eye‐tracking studies revealed that greater attention to socially relevant stimuli, such as faces and eyes, is associated with better social functioning and reduced autism symptom severity in autistic individuals across a broad developmental span (Riddiford et al. 2022). Similarly, another meta‐analysis revealed associations between gaze behaviors reflecting different attention constructs (e.g., social attention, joint attention) and social communication skills among young autistic children (Yoon et al. 2025). These findings bolster the utility of eye tracking as a meaningful indicator of symptom severity within the social communication domain. Nevertheless, the existing body of evidence predominantly comprises cross‐sectional studies that focus on concurrent associations rather than longitudinal associations. This narrow perspective creates a notable gap in understanding how eye‐tracking measures can detect incremental improvements, evaluate intervention efficacy, and predict outcomes over time. Addressing this gap is essential for leveraging eye tracking as a tool for treatment evaluation. To this end, we propose two distinct frameworks for examining the short‐term and long‐term effects of autism treatments: treatment monitoring and prediction of treatment response.

A substantial body of research has demonstrated systematic differences in visual attention in autistic individuals compared to neurotypical controls, including diminished spontaneous attention to faces (Shic et al. 2020, 2023), reduced gaze following (de Belen et al. 2023; Wang et al. 2020), and attenuated attentional disengagement (Sacrey et al. 2014). These atypical attentional patterns are widely regarded as fundamental to downstream challenges in social communication (Dawson et al. 2004; Mundy and Crowson 1997; Riddiford et al. 2022; Yoon et al. 2025). Given that these constructs are well‐characterized and differentiated from neurotypical controls, treatment‐induced changes in eye‐tracking measures hold clear significance. Changes in attention allocation or duration may not merely reflect changes in looking time but instead could also indicate the emergence of, or divergence from, more typical attentional processes, depending on the direction of changes and the attentional construct being quantified. In this manner, eye‐tracking measures provide a concrete means of linking intervention effects to underlying alterations in attentional mechanisms, serving as a readily interpretable and mechanistically meaningful indicator of treatment impact.

Consequently, emerging autism intervention studies—including behavioral, pharmacological, and technology‐based approaches—have examined eye‐tracking outcomes as a treatment‐sensitive biomarker. These studies have documented changes such as increased fixation on faces or inner facial features (e.g., Amat et al. 2021; Billeci et al. 2017; Dawson et al. 2017; Gepner et al. 2022; Tang et al. 2022), which often differ in autistic individuals compared to neurotypical controls (Shic et al. 2020, 2023). Notably, these changes occur even in interventions, such as speech therapy, that do not explicitly target gaze (e.g., Gepner et al. 2022), suggesting that eye tracking can capture broader developmental adjustments and indirect effects beyond the immediate targets of the intervention. It is, however, essential to recognize that not all studies have shown significant changes in eye‐tracking outcomes post‐treatment (e.g., Scherf et al. 2024; Wong, Tan, Koh, et al. 2024). These null findings provide important interpretive context. While the absence of measurable changes could stem from methodological factors such as study design and participant characteristics, it may also reflect state‐related influences (e.g., fatigue, anxiety, or situational factors) that naturally introduce intraindividual variability. In contrast, consistent changes are more likely to indicate true changes in trait‐like aspects of gaze behavior rather than transient noise. Thus, both stability and change contribute to interpretation, positioning eye tracking as a valuable tool for monitoring treatment.

The utility of eye tracking as both a prognostic and predictive biomarker has also been evident in autism research. A prognostic biomarker refers to an indicator that predicts the likely course of a condition independent of treatment (Califf 2018), whereas a predictive biomarker refers to an indicator that predicts the likelihood of response to a specific treatment (Califf 2018). Longitudinal studies outside of intervention contexts have shown that early differences in social attention—such as reduced spontaneous attention to faces, diminished monitoring of social scenes, or a stronger preference for nonsocial geometric patterns—can predict later autism symptom severity (Bacon et al. 2020; Chawarska et al. 2013; Shic et al. 2014), social functioning (Bacon et al. 2020), and adaptive skills (Bacon et al. 2020). These findings suggest that eye tracking captures enduring individual differences in attentional patterns that are linked to long‐term developmental outcomes, highlighting its prognostic value.

Accordingly, intervention studies have begun to investigate its predictive utility by examining whether initial eye‐tracking profiles can predict treatment responses in autistic individuals, primarily in children (e.g., Bent et al. 2023; Greene et al. 2021; Robain et al. 2020). These studies demonstrate that autistic children who begin intervention with higher levels of social attention—such as spending more time looking at faces or orienting more readily to social cues—achieve greater progress across developmental domains such as language, cognitive, social, and adaptive functioning. One study further suggests that eye‐tracking outcomes reflecting learning processes can predict developmental gains even when social attention does not (Vivanti et al. 2013). Specifically, Vivanti et al. demonstrated that eye‐tracking measures of goal understanding predict 1‐year receptive language gains in autistic children receiving the Early Start Denver Model intervention (ESDM; Rogers and Dawson 2020), while eye‐tracking measures of social attention (overall face‐looking) showed no association with these outcomes. These findings suggest that eye‐tracking data collected at initial assessment may not merely reflect transient gaze patterns but index enduring attentional and learning tendencies that shape how children perceive and assimilate socially relevant information. Because these tendencies facilitate access to and use of learning opportunities embedded within the intervention, baseline eye‐tracking outcomes may elucidate mechanisms underlying individual differences in developmental outcomes following intervention.

Both treatment monitoring and prediction of treatment response frameworks highlight the potential of eye tracking for longitudinal assessment. Nonetheless, the varying strength and consistency require a better understanding of the overall scope of the effect. Thus, this meta‐analysis aims to estimate the summary effect size of changes in eye‐tracking outcomes from pre‐ to post‐treatment in autistic individuals and to examine methodological factors (e.g., study design, participant characteristics) that may account for observed variations. Additionally, the exploratory aim is to estimate the summary effect size of the predictive association between baseline (pre‐treatment) eye‐tracking outcomes and developmental changes from pre‐ to post‐treatment in autistic individuals, along with an assessment of methodological factors. Ultimately, this work seeks to advance our understanding of how objective eye‐tracking data can inform more effective and personalized strategies for autism intervention and assessment.

## 1 Method

**FIGURE 1:**
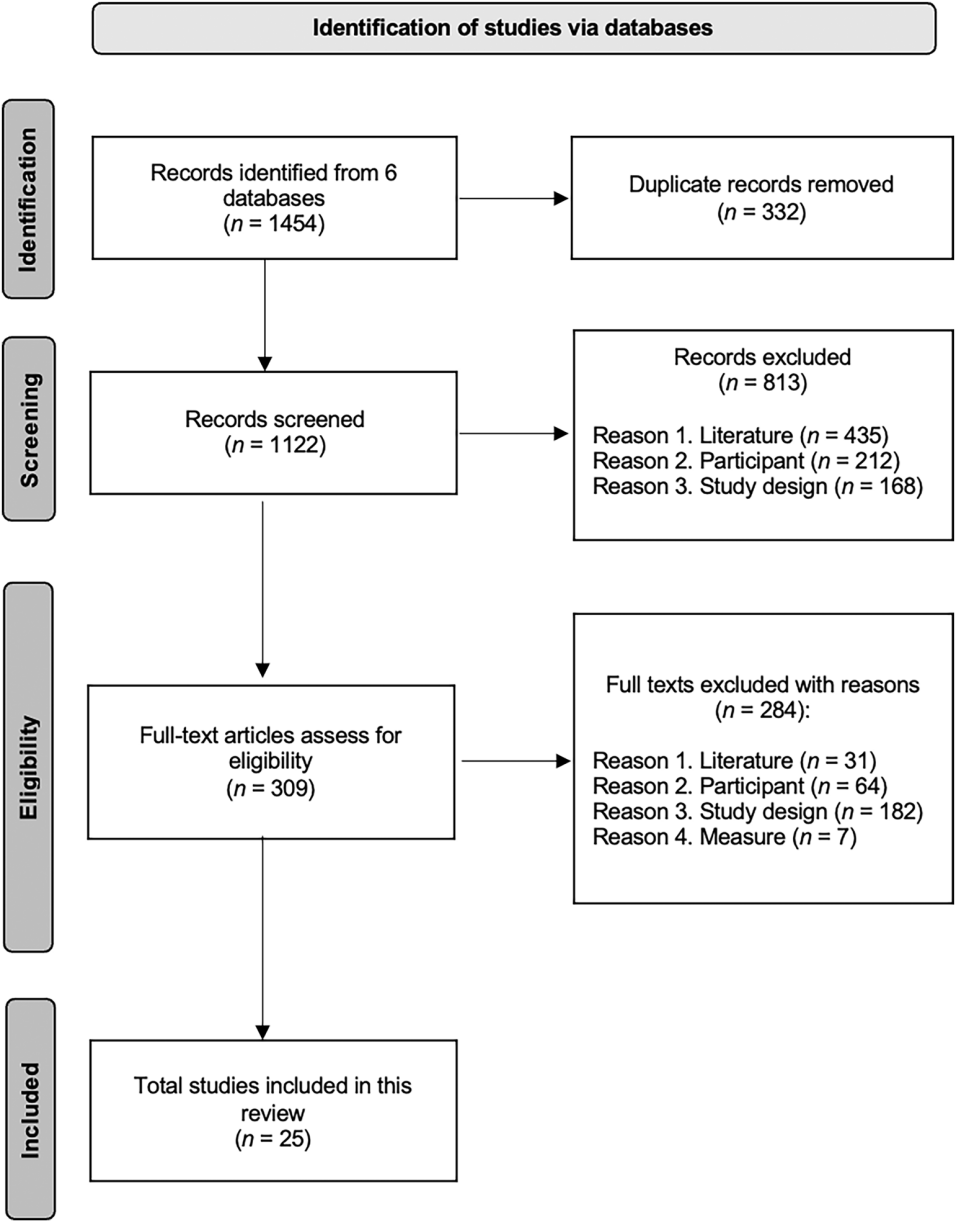
PRISMA flow chart.

We adhered to the Preferred Reporting Items for Systematic Reviews and Meta‐Analyses Statement (PRISMA; Page et al. 2021). The search strategy, selection process, and results are illustrated in the PRISMA flowchart in Figure 1.

### 1.1 Study Selection

#### 1.1.1 Eligibility Criteria

Articles were included if they met the following criteria: (a) literature—published in a peer‐reviewed journal, empirical, and written in English; (b) participants—included all or at least one group of participants with autism; (c) study design—used a pre‐post‐treatment design with eye tracking conducted before and/or after the intervention; and (d) measures—reported effect sizes (e.g., Cohen's *d*) related to changes in eye‐tracking outcomes from pre‐ to post‐treatment, correlation coefficients between baseline eye‐tracking outcomes and changes in developmental outcomes from pre‐ to post‐treatment, or other statistics (e.g., means, SD) to calculate an effect size. No restrictions were placed on publication date, participant age, or treatment type.

#### 1.1.2 Search Process

A search was conducted in October 2023 and April 2025 across six databases: Academic Search Ultimate, ERIC, MEDLINE, PsycArticles, PsycINFO, and PubMed. The following keywords related to the areas of interest were used: (a) autism (“*autism*” OR “ASD” OR “autism spectrum disorder” OR “autis*”); (b) eye tracking (“*eye tracking*” OR “*eye‐tracking*” OR “*eye‐track*” OR “*gaze*” OR “*gaze behavior*”); and (c) treatment response (“*treatment*” OR “*treatment outcome*” OR “*treatment response*” OR “*predictor*” OR “*prognostic*”). The initial search retrieved a total of 1454 articles. After removing duplicates, 1122 articles remained for screening.

#### 1.1.3 Screening Process

The articles were assessed in two stages. First, the titles and abstracts of the 1122 articles were screened against the eligibility criteria; 813 articles were excluded. Subsequently, the full text of the remaining 309 articles was reviewed, resulting in the removal of 284 articles. As a result, 25 articles were included in the study.

### 1.2 Data Evaluation

Effect sizes indicating changes in eye‐tracking outcomes from pre‐ to post‐treatment, the correlation between baseline eye‐tracking outcomes and changes in developmental outcomes from pre‐ to post‐treatment, or other statistics used to calculate an effect size were extracted from each study. Additionally, the following variables for each effect size were collected: participant characteristics (sample size, mean age, sex), study design (randomization, blinding, dropout rate), treatment (type, duration), the motion format of the eye‐tracking stimulus (static, dynamic), derived gaze metrics, and clinical assessments used to evaluate changes in developmental domains following treatment.

#### 1.2.1 Standardizing Effect Sizes

We used different standardization methods because our dataset included two distinct types of effect sizes: mean‐change and correlational outcomes. For effect sizes indicating changes in eye‐tracking outcomes from pre‐ to post‐treatment, when values were not reported directly, we calculated Cohen's *d* from reported statistics (e.g., means, standard deviations). Then, we corrected for small‐sample bias by converting Cohen's *d* to Hedges' *g* (Hedges 1981). For effect sizes indicating correlation between baseline eye‐tracking outcomes and changes in developmental outcomes from pre‐ to post‐treatment, we first adjusted the direction of correlation coefficients with opposing signs that nonetheless reflected the same underlying relationship. For example, a negative correlation could indicate either more or less positive developmental outcomes depending on the assessment used. This approach ensured all effect sizes aligned with a consistent interpretation. Other reported statistics (e.g., partial eta‐squared) were also converted into correlation coefficients when possible. Finally, to meet the assumptions of normality and stabilize variance, we applied Fisher's *r*‐to‐*z* transformation to all correlation coefficients (Fisher 1921).

#### 1.2.2 Moderators

Eight moderators were examined for their potential impact on changes in eye‐tracking outcomes from pre‐ to post‐treatment. These included publication year, participants' age (mean in years), participants' sex (% male), treatment duration (in weeks), randomization status, blinding status, treatment type, and the motion format of the eye‐tracking stimulus. Randomization status was categorized as either randomized (i.e., randomly assigned to treatment or control groups) or non‐randomized (i.e., assigned based on predetermined criteria or other non‐random factors, such as single‐arm trials). Blinding status was classified as either blinded (i.e., autistic participants unaware of the active treatment) or non‐blinded (i.e., autistic participants fully aware of their treatment assignment). Treatment type was divided into three broad categories based on intervention features: behavioral treatment (e.g., ESDM) targeting social communication and adaptive functioning, biomedical treatment (e.g., nasal oxytocin or blood infusion) focusing on the social brain, and technology‐based treatment (e.g., game‐based interventions) targeting attention. Lastly, the motion format was categorized as dynamic stimuli (i.e., moving formats like videos) or static stimuli (i.e., non‐moving formats like still images).

Five moderators were examined for their potential impact on the predictive association between baseline eye‐tracking outcomes and changes in developmental outcomes following treatment. These included: publication year, participants' age (mean in years), participants' sex (% male), treatment duration (in weeks), and developmental domain. Specifically, the developmental domain was categorized into two groups based on how outcomes were defined and measured in the included studies: behavioral domain, represented by measures such as ADOS and VABS, and cognitive domain, indicated by measures such as MSEL and developmental quotient scores. See Table S1 for an explanation of moderator selection.

#### 1.2.3 Inter‐Rater Reliability

Inter‐rater reliability was assessed between two coders, who evaluated all data or 28% (*n* = 7) of the 25 articles, respectively. Reliability was determined by the percentage of variables that were consistent between both coders for each effect size. It ranged from 82% to 100%, with an average of 96%. Any disagreements were resolved through consensus discussions, resulting in a final, mutually agreed‐upon judgment for each variable.

### 1.3 Quality Assessment

The quality of 25 studies was assessed using Cochrane‐recommended tools based on their design: RoB 2.0 for randomized studies (Sterne et al. 2019) and ROBINS‐I for non‐randomized studies (Sterne et al. 2016). Since our meta‐analysis focused on within‐group pre‐post changes in autistic participants, we modified the ROBINS‐I tool to assess the confounding domain by determining whether studies indicated a within‐group change signal. For the outcome measurement domain (included in both tools), we assessed studies reporting pre‐post eye‐tracking outcomes, emphasizing the objectivity and validity of the data, following standard guidance for studies reporting correlations between baseline eye‐tracking outcomes and changes in clinical assessments. All other domains were rated according to standard guidance (Tables S2 and S3).

### 1.4 Statistical Analysis

#### 1.4.1 Model Specification for Meta‐Analysis

We conducted a multivariate random‐effects meta‐analysis with a three‐level structure (Assink and Wibbelink 2016; Cheung 2014, 2019) in R version 4.4.0 (R Core Team 2024) using the “*metafor*” package (Viechtbauer 2010). This model partitions the total variance into three components: sampling error associated with each effect size, within‐study heterogeneity capturing variability among multiple effect sizes reported within the same study, and between‐study heterogeneity reflecting variability across studies. To assess the robustness of our results, we conducted sensitivity analyses across three plausible intra‐cluster correlation assumptions (*ρ* = 0.3, 0.6, and 0.9).

#### 1.4.2 Bias and Influential Effects

A comprehensive approach was adopted to assess potential sources of bias, including small‐study effects and influential cases. Initially, we incorporated the standard error (SE) as a predictor within the multilevel model to statistically test for funnel plot asymmetry (i.e., small‐study effects), evaluating whether smaller studies with greater variability systematically exhibited different effect sizes. Subsequently, we visually inspected the funnel plot (Light and Pillemer 1984), with asymmetry suggesting a potential presence of publication bias. Finally, we used Cook's distance (Cook 2000) to identify influential effect sizes that disproportionately impacted the model, providing insights into outliers that may skew the results. When potential bias or influential cases were identified, we re‐estimated the summary effect size across different intra‐cluster correlation assumptions (*ρ* = 0.3, 0.6, and 0.9) to ascertain stability.

#### 1.4.3 Subgroup and Moderation Analyses

Subgroup and moderation analyses were conducted within the same multivariate three‐level model specification. First, we performed subgroup analyses for four categorical moderators (treatment randomization status, treatment blinding status, treatment type, and stimulus motion format) to estimate changes in eye‐tracking outcomes from pre‐ to post‐treatment within each subgroup. Then, we conducted meta‐regression to formally test whether the summary effect sizes differ significantly across these subgroups and to evaluate continuous moderators, analyzing each moderator separately to determine its unique contribution. All subgroup and moderation analyses were carried out after addressing potential outliers to minimize the risk of biased results. A single *ρ* value (0.6) was used for moderation analyses, based on the stability of the findings.

## 2 Results

A total of 218 effect sizes were included in the analyses, comprising (a) 179 effect sizes for changes in eye‐tracking outcomes from pre‐ to post‐treatment, and (b) 39 effect sizes for the correlation between baseline eye‐tracking outcomes and changes in developmental outcomes from pre‐ to post‐treatment. These effect sizes involve 828 autistic participants, with a mean age ranging from 3 to 28 years. Our analyses focused solely on autistic participants for this study. See Tables S4 and S5 for study information.

### 2.1 Changes in Eye‐Tracking Outcomes From Pre‐ to Post‐Treatment

#### 2.1.1 Summary Effect Size

**FIGURE 2:**
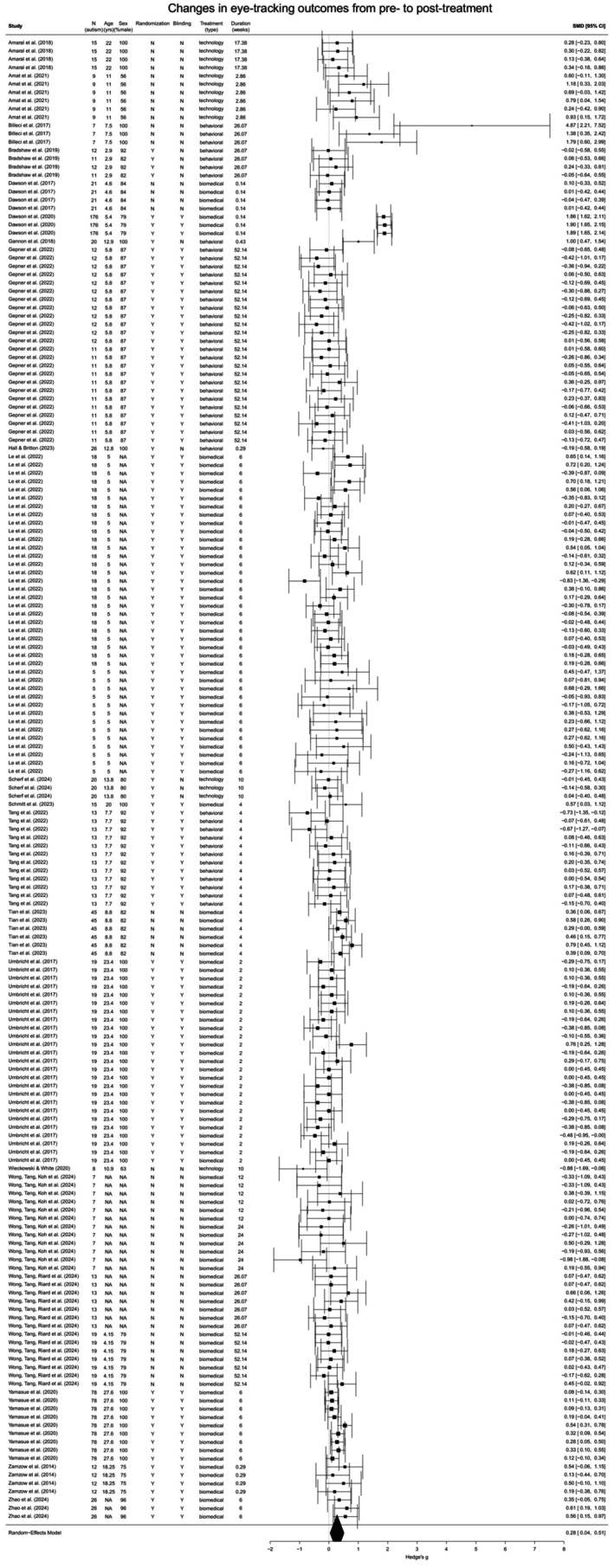
Forest plot for changes in eye‐tracking outcomes from pre‐ to post‐treatment in autistic individuals. *Note*. For Wong, Tan, Riard, et al. (2024), the treatment duration refers to the period from a one‐time treatment to follow‐up. Y = yes; N = no.

**FIGURE 3:**
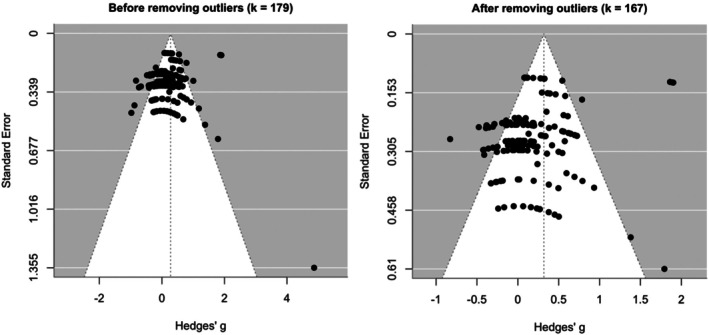
Funnel plots for changes in eye‐tracking outcomes from pre‐ to post‐treatment.

The analysis revealed a modest, significant summary effect size for changes in eye‐tracking outcomes from pre‐ to post‐treatment (*ρ* = 0.3, Hedge's *g* = 0.29, 95% CI [0.05, 0.53], *p* = 0.018, *I* ^*2*^ = 75.0%; *ρ* = 0.6, Hedge's *g* = 0.28, 95% CI [0.04, 0.51], *p* = 0.021, *I* ^*2*^ = 74.2%; and *ρ* = 0.9, Hedge's *g* = 0.25, 95% CI [0.02, 0.49], *p* = 0.032, *I* ^*2*^ = 74.2%). See Figure 2 for the forest plot illustrating both individual and summary effect sizes. An assessment of potential bias indicated no significant small‐study effects (*b* = 1.06, 95% CI [−0.18, 2.30], *p* = 0.092); however, the funnel plot showed notable asymmetry (Figure 3), and influential effect sizes (*k* = 12) were identified (Figure S1).

Re‐estimating without these influential effect sizes increased the summary effect size for changes in eye‐tracking outcomes from pre‐ to post‐treatment (*ρ* = 0.3, Hedge's *g* = 0.33, 95% CI [0.08, 0.57], *p* = 0.009, *I* ^*2*^ = 75.5%; *ρ* = 0.6, Hedge's *g* = 0.32, 95% CI [0.08, 0.57], *p* = 0.010, *I* ^*2*^ = 75.8%; and *ρ* = 0.9, Hedge's *g* = 0.32, 95% CI [0.07, 0.57], *p* = 0.012, *I* ^2^ = 76.4%), which remained within a moderate range and statistically significant, consistent with the initial estimate. The symmetry of the funnel plot also improved (Figures 3 and S2). These results suggest that outliers may have dampened the initial estimate and that the revised estimate likely provides a more precise summary of changes in eye‐tracking outcomes post‐treatment.

#### 2.1.2 Subgroup Differences and Moderating Effects

Subgroup analysis based on randomization status revealed a moderate, significant summary effect size for the non‐randomized group (*ρ* = 0.3, Hedge's *g* = 0.30, 95% CI [0.05, 0.54], *p* = 0.019; *ρ* = 0.6, Hedge's *g* = 0.27, 95% CI [0.05, 0.49], *p* = 0.016; *ρ* = 0.9, Hedge's *g* = 0.26, 95% CI [0.04, 0.48], *p* = 0.018), while it revealed a moderate but non‐significant summary effect size for the randomized group (*ρ* = 0.3, Hedge's *g* = 0.31, 95% CI [−0.04, 0.67], *p* = 0.079; *ρ* = 0.6, Hedge's *g* = 0.32, 95% CI [−0.04, 0.67], *p* = 0.080; *ρ* = 0.9, Hedge's *g* = 0.32, 95% CI [−0.04, 0.68], *p* = 0.081). However, randomization status revealed no moderating effect (*Q*_*M*_ = 0.003, *p* = 0.958), suggesting no difference in changes in eye‐tracking outcomes post‐treatment between randomized and non‐randomized groups, and that pooling of the samples is appropriate.

Similarly, subgroup analysis based on blinding status revealed a moderate, significant summary effect size for the non‐blinded group (*ρ* = 0.3, Hedge's *g* = 0.22, 95% CI [0.03, 0.41], *p* = 0.022; *ρ* = 0.6, Hedge's *g* = 0.21, 95% CI [0.03, 0.39], *p* = 0.025; *ρ* = 0.9, Hedge's *g* = 0.20, 95% CI [0.02, 0.39], *p* = 0.032), while it revealed a moderate but non‐significant summary effect size for the blinded group (*ρ* = 0.3, Hedge's *g* = 0.38, 95% CI [−0.04, 0.80], *p* = 0.073; *ρ* = 0.6, Hedge's *g* = 0.38, 95% CI [−0.04, 0.80], *p* = 0.074; *ρ* = 0.9, Hedge's *g* = 0.38, 95% CI [−0.04, 0.81], *p* = 0.075). However, blinding status revealed no moderating effect (*Q*_*M*_ = 0.25, *p* = 0.620), suggesting no difference in changes in eye‐tracking outcomes post‐treatment between blinded and non‐blinded groups, and that pooling of the samples is appropriate.

Subgroup analysis based on treatment type revealed a moderate, significant summary effect size for the biomedical treatment group (*ρ* = 0.3, Hedge's *g* = 0.37, 95% CI [0.03, 0.71], *p* = 0.036; *ρ* = 0.6, Hedge's *g* = 0.37, 95% CI [0.02, 0.71], *p* = 0.037; *ρ* = 0.9, Hedge's *g* = 0.37, 95% CI [0.02, 0.71], *p* = 0.039), while it revealed a moderate but non‐significant summary effect size for both the technology‐based (*ρ* = 0.3, Hedge's *g* = 0.24, 95% CI [−0.11, 0.58], *p* = 0.175; *ρ* = 0.6, Hedge's *g* = 0.19, 95% CI [−0.11, 0.50], *p* = 0.219; *ρ* = 0.9, Hedge's *g* = 0.14, 95% CI [−0.16, 0.45], *p* = 0.347) and behavioral treatment groups (*ρ* = 0.3, Hedge's *g* = 0.28, 95% CI [−0.34, 0.90], *p* = 0.373; *ρ* = 0.6, Hedge's *g* = 0.25, 95% CI [−0.32, 0.81], *p* = 0.389; *ρ* = 0.9, Hedge's *g* = 0.11, 95% CI [−0.20, 0.41], *p* = 0.496). However, treatment type revealed no moderating effect (*Q*_*M*_ = 0.18, *p* = 0.914), suggesting no difference in changes in eye‐tracking outcomes post‐treatment between autistic individuals receiving different types of treatment, and that pooling of the samples is appropriate.

Subgroup analysis based on eye‐tracking stimulus motion format revealed a moderate, significant summary effect size for dynamic stimuli (*ρ* = 0.3, Hedge's *g* = 0.36, 95% CI [0.09, 0.63], *p* = 0.010; *ρ* = 0.6, Hedge's *g* = 0.35, 95% CI [0.08, 0.63], *p* = 0.012; *ρ* = 0.9, Hedge's *g* = 0.35, 95% CI [0.07, 0.63], *p* = 0.015), while it revealed a moderate but non‐significant summary effect size for static stimuli (*ρ* = 0.3, Hedge's *g* = 0.14, 95% CI [−0.08, 0.36], *p* = 0.202; *ρ* = 0.6, Hedge's *g* = 0.14, 95% CI [−0.07, 0.36], *p* = 0.198; *ρ* = 0.9, Hedge's *g* = 0.14, 95% CI [−0.07, 0.35], *p* = 0.195). However, motion format revealed no moderating effect (*Q*_*M*_ = 3.37, *p* = 0.066), suggesting no difference in changes in eye‐tracking outcomes post‐treatment between using dynamic and static stimuli, and that pooling of the samples is appropriate.

Lastly, the summary effect size for changes in eye‐tracking outcomes from pre‐ to post‐treatment was not significantly moderated by publication year (*b* = −0.02, 95% CI [−0.11, 0.06], *p* = 0.594), age (*b* = −0.01, 95% CI [−0.04, 0.03], *p* = 0.637), sex (*b* = −0.001, 95% CI [−0.02, 0.02], *p* = 0.910) or treatment duration (*b* = −0.002, 95% CI [−0.01, 0.01], *p* = 0.613).

### 2.2 Correlation Between Baseline Eye‐Tracking Outcomes and Changes in Developmental Outcomes From Pre‐ to Post‐Treatment

#### 2.2.1 Summary Effect Size

**FIGURE 4:**
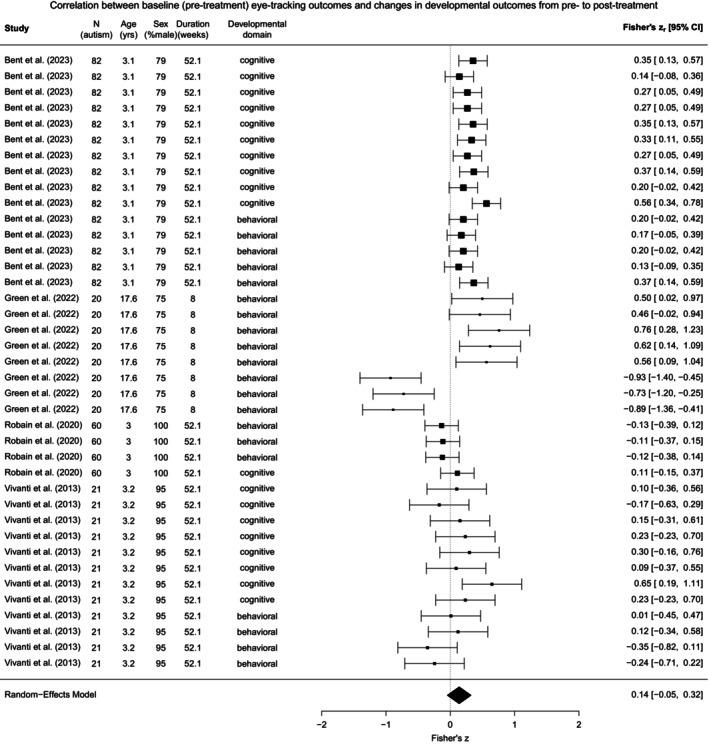
Forest plot for the correlation between baseline (pre‐treatment) eye‐tracking outcomes and changes in developmental outcomes from pre‐ to post‐treatment in autistic individuals.

**FIGURE 5:**
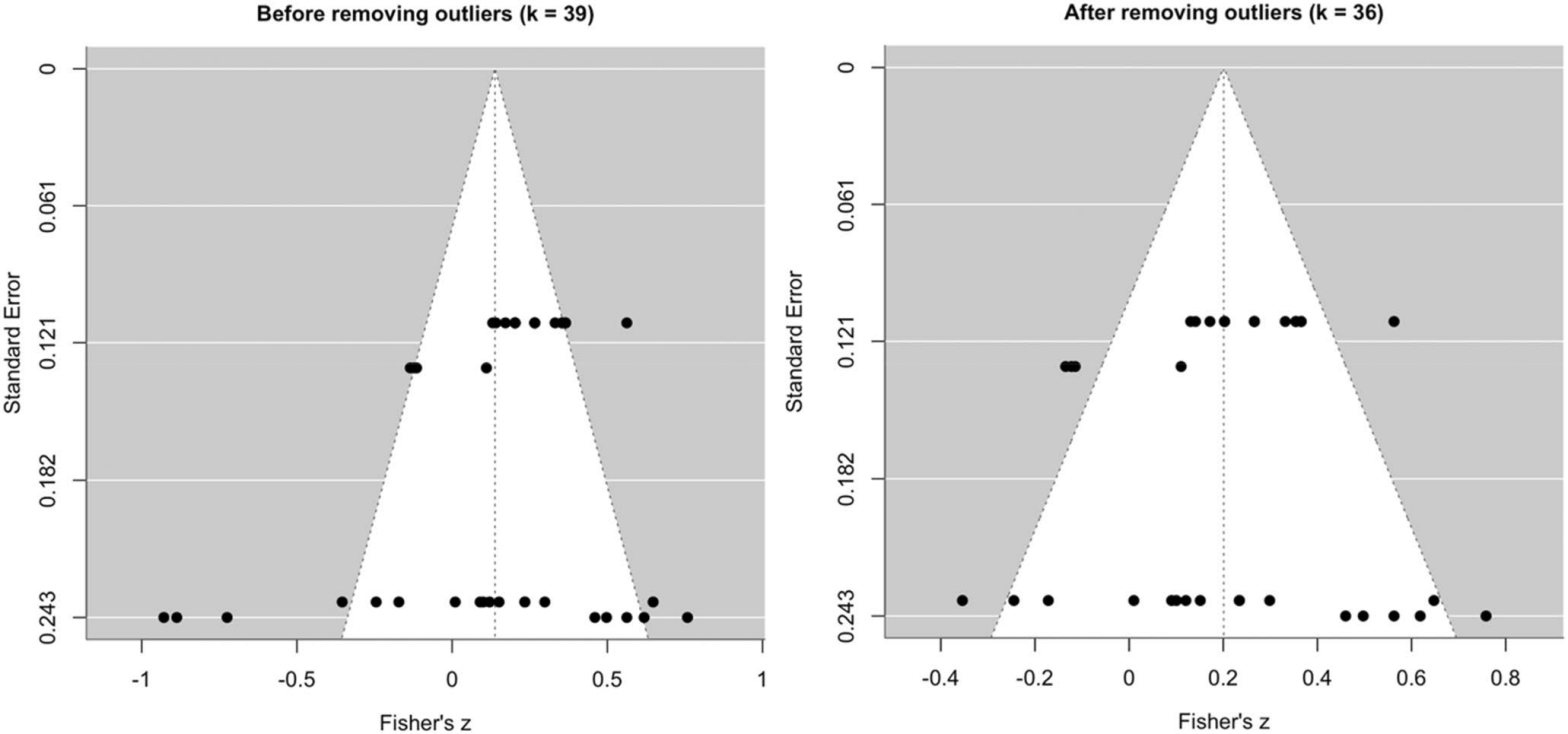
Funnel plots for the correlation between baseline (pre‐treatment) eye‐tracking outcomes and changes in developmental outcomes from pre‐ to post‐treatment.

The analysis revealed a moderate, non‐significant summary effect size for correlation between baseline eye‐tracking outcomes and changes in developmental outcomes from pre‐ to post‐treatment (*ρ* = 0.3, Fisher's *z* = 0.13, 95% CI [−0.05, 0.30], *p* = 0.146, *I* ^*2*^ = 72.0%; *ρ* = 0.6, Fisher's *z* = 0.14, 95% CI [−0.05, 0.32], *p* = 0.142, *I* ^*2*^ = 75.8%; and *ρ* = 0.9, Fisher's *z* = 0.15, 95% CI [−0.05, 0.34], *p* = 0.145, *I* ^*2*^ = 78.6%). See Figure 4 for the forest plot illustrating both individual and summary effect sizes. An assessment for potential bias indicated no significant small‐study effects (*b* = −1.13, 95% CI [−4.36, 2.10], *p* = 0.493) or notable asymmetry in the funnel plot (Figure 5). However, influential effect sizes were identified (*k* = 3; Figure S1).

Re‐estimating without these influential effect sizes increased the summary effect size for correlation between baseline eye‐tracking outcomes and changes in developmental outcomes from pre‐ to post‐treatment (*ρ* = 0.3, Fisher's *z* = 0.21, 95% CI [−0.05, 0.46], *p* = 0.112, *I* ^*2*^ = 63.3%; *ρ* = 0.6, Fisher's *z* = 0.20, 95% CI [−0.05, 0.45], *p* = 0.115, *I* ^*2*^ = 61.8%; and *ρ* = 0.9, Fisher's *z* = 0.20, 95% CI [−0.05, 0.44], *p* = 0.114, *I* ^*2*^ = 63.1%), which remained within a moderate range and non‐significant. The symmetry of the funnel plot also improved (Figures 5 and S3). These results suggest that outliers may have dampened the initial estimate and that the revised estimate likely provides a more precise summary of the predictive association.

#### 2.2.2 Moderating Effects

**FIGURE 6:**
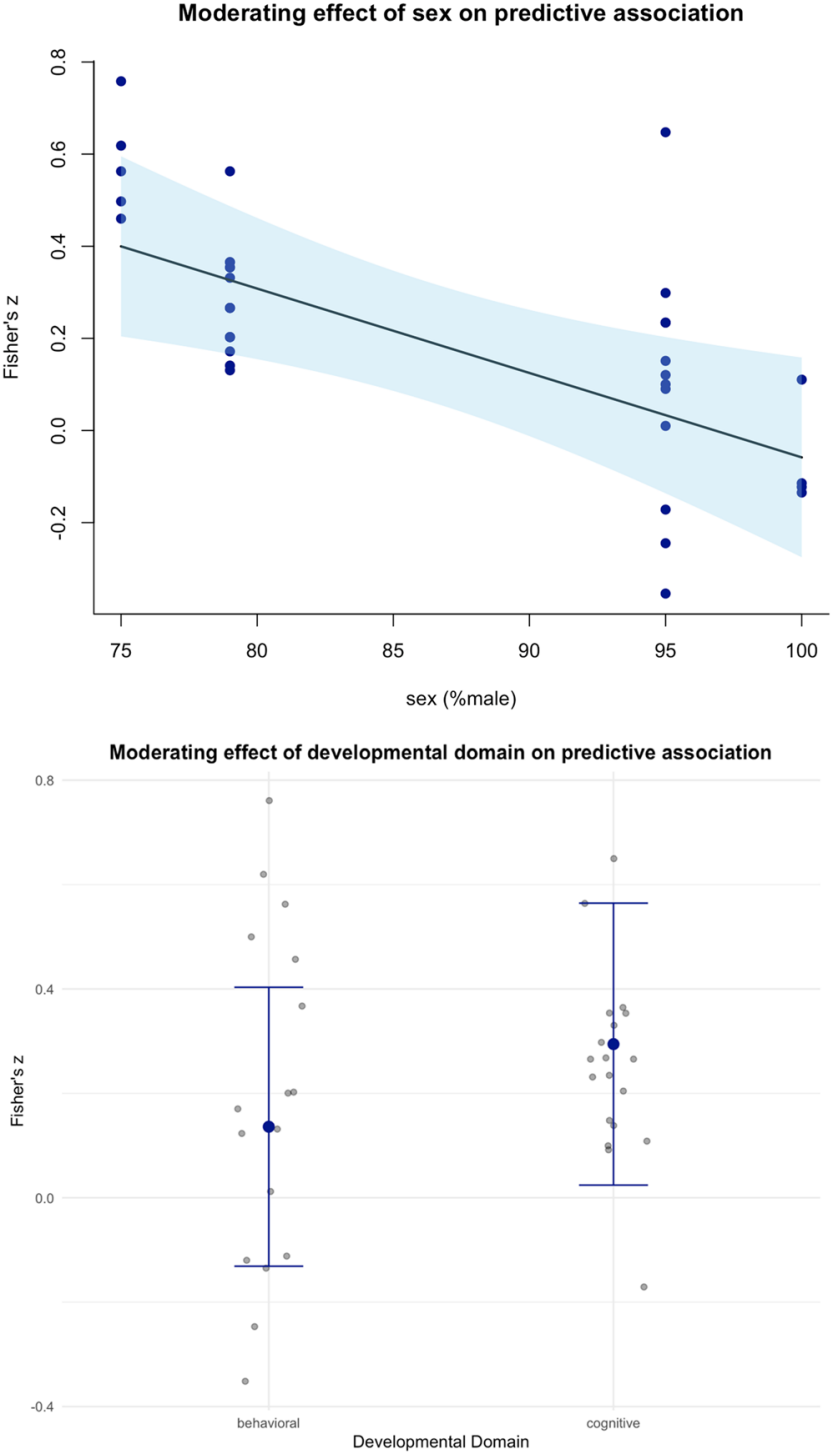
Moderating effects on the predictive association.

The summary effect size for the correlation between baseline eye‐tracking outcomes and changes in developmental outcomes from pre‐ to post‐treatment was significantly moderated by sex (*b* = −0.02, 95% CI [−0.03, −0.01], *p* = 0.005), suggesting that a higher proportion of male autistic participants weakens the predictive association between baseline eye‐tracking profiles and changes in developmental domains in response to treatment (Figure 6). The specific developmental domain that changed from pre‐ to post‐treatment also contributed to variations in the summary effect size (*Q*_*M*_ = 10.18, *p* = 0.001), showing a stronger association with changes in the cognitive domain (*b* = 0.16, 95% CI [0.06, 0.26], *p* = 0.001) than with changes in the behavioral domain (Figure 6). However, the summary effect size was not moderated by publication year (*b* = 0.03, 95% CI [−0.05, 0.10], *p* = 0.464), age (*b* = 0.03, 95% CI [−0.01, 0.07], *p* = 0.097), or treatment duration (*b* = −0.01, 95% CI [−0.02, 0.002], *p* = 0.100).

## 3 Discussion

This meta‐analysis investigated two distinct applications of eye tracking to enhance understanding of its efficacy in evaluating both short‐term and long‐term treatment outcomes in autistic individuals: treatment monitoring and prediction of treatment response.

### 3.1 Eye Tracking as a Treatment Monitoring Tool

This work revealed a small‐to‐moderate summary effect size concerning pre‐ to post‐treatment changes in eye‐tracking outcomes among autistic individuals. Notably, a formal minimal clinically important difference (MCID; Jaeschke et al. 1989) has not yet been established for eye‐tracking biomarkers in autism. Nevertheless, MCID estimates from a large dataset of over 9000 autistic individuals for VABS‐II—the most widely used tool for assessing adaptive behaviors essential for socialization, communication, and daily functioning—serve as a valuable reference point. An MCID for VABS‐II suggests that a change of 2 to 3.75 points on its composite score indicates clinically meaningful improvement (Chatham et al. 2018). Although these benchmarks cannot be directly applied to eye‐tracking measures, they roughly correspond to a standardized change of about 0.13–0.25 standard deviations, thereby providing a conceptual basis: a small‐to‐moderate effect size observed in this study (0.33–0.32 across assumptions) likely reflects a reliable and measurable group‐level change, albeit incremental rather than transformative. While this suggests that interventions appear capable of changing attentional patterns in autism, it remains uncertain if such changes translate into functional gains in daily social communication, adaptive behavior, or overall quality of life, unless these changes are supported by interventions that facilitate generalization.

The subgroup findings introduce an important layer of complexity to interpretation. Although patterns of significance varied across groups (randomization status, blinding status, treatment type, and stimulus motion format), the corresponding moderator tests were non‐significant, suggesting that apparent subgroup differences should be interpreted with caution. A significant change in eye‐tracking outcomes was observed in both non‐randomized and non‐blinded groups of autistic individuals, whereas randomized and blinded groups did not show the same pre‐post change. This divergence aligns with meta‐analytic evidence showing that intervention effects in autism research become much smaller—and in some cases disappear—when analyses are restricted to randomized trials with independent assessors (Sandbank et al. 2020). Such discrepancies raise critical questions about expectancy effects, caregiver engagement, and contextual factors surrounding intervention delivery. Less controlled studies create conditions in which awareness of active treatment can heighten participant motivation, increase caregiver involvement, and amplify therapist enthusiasm—all factors that could shape the social contingencies of the intervention environment. Such expectancy‐related biases have been identified for their role in inflating apparent treatment in autism intervention research (Bottema‐Beutel 2023; Sandbank et al. 2020). Because autistic individuals' gaze behavior is highly sensitive to social context (Chawarska et al. 2012) and the perceived responsiveness (Wang et al. 2020), these expectancy‐associated dynamics may yield measurable changes in eye‐tracking indices. Blinded designs dampen these cues, producing more conservative, and arguably more accurate, estimates of treatment‐driven change. Taken together, this pattern suggests that the observed eye‐tracking outcomes in this work may reflect both neurocognitive shifts and contextual influences related to expectancy and engagement.

Subgroup patterns by treatment type further illustrate this point. Autistic individuals receiving biomedical interventions exhibited a significant change in eye‐tracking outcomes, whereas those undergoing behavioral or technology‐based treatments did not. This disparity aligns with established knowledge regarding intervention mechanisms. Biomedical treatments, such as pharmacological agents, may directly modulate neural circuits that govern visual attention and contribute to the regulation of social attention (e.g., dopaminergic and oxytocinergic systems; Lockhofen and Mulert 2021), potentially inducing rapid changes that are detectable through eye‐tracking technology. Conversely, behavioral interventions typically target broader, complex developmental skills, such as communication, social reciprocity, and adaptive functioning, which may lead to more diffuse or incremental changes in visual attention. Consequently, their effects on attentional patterns may occur indirectly and over extended periods, making immediate and measurable changes in gaze less likely within the often less than six‐month pre–post intervention trials (Grzadzinski et al. 2020). Interestingly, although technology‐based interventions often directly target attentional processes by incorporating dynamic, visually stimulating content designed to engage attention effectively (Vedechkina and Borgonovi 2021), such features may predominantly engage bottom‐up, reactive attention mechanisms. This reliance on reactive processes could account for the absence of significant changes in eye‐tracking outcomes.

Findings further revealed that dynamic stimuli (e.g., videos) elicited a significant treatment‐related change in eye‐tracking outcomes in autistic individuals, whereas static stimuli (e.g., images) did not. This aligns with evidence suggesting that gaze differences in autistic individuals, particularly children, are more pronounced when viewing moving, socially engaging scenes (Chevallier et al. 2015; Cilia et al. 2019; Saitovitch et al. 2013; Speer et al. 2007). This is presumably because such stimuli more closely mirror the demands of social interaction that often pose challenges for autistic individuals. By tapping into these real‐time processing demands, dynamic formats may heighten the visibility of social processing differences targeted by interventions. This, in turn, enhances their sensitivity compared to static stimuli for detecting both fundamental gaze differences and subtle, treatment‐related changes over time. Nevertheless, it is imperative to note that none of the subgroup distinctions, including randomization status, blinding status, treatment type, and stimulus motion format, demonstrated statistical significance between groups. This suggests that the observed subgroup patterns may be attributable to study‐level variability, such as unequal representation of intervention types or heterogeneity in methodologies, rather than definitive categorical distinctions.

Equally important are the nonsignificant moderators, including publication year, participant age, sex, and treatment duration. The absence of moderation by publication year suggests that the effects of treatment on eye‐tracking outcomes have remained stable from 2014 (Zamzow et al. 2014) to 2024 (e.g., Zhao et al. 2024). This temporal consistency reinforces the robustness of eye tracking as a biomarker, while highlighting the necessity for continued innovation to enhance its sensitivity. Increased sensitivity would enable these measures to detect subtle, domain‐specific changes, especially in core autism symptoms such as social communication difficulties, which may be limited by tools oriented toward long‐term developmental milestones (Grzadzinski et al. 2020; Rogers et al. 2012; Thurm et al. 2015).

The absence of age moderation suggests that eye‐tracking outcomes are consistent across developmental stages, reflecting the stability of the fundamental mechanisms underlying visual attention in autism (Frazier et al. 2017). This consistency is significant as it supports the use of eye‐tracking measures as a universal tool for evaluating treatment effects, independent of participants' ages. Nonetheless, this stability may also reflect an implicit alignment of measures with developmental appropriateness rather than true universality across age groups. Consequently, it remains unclear whether these measures are inherently insensitive to developmental nuances or if their apparent stability results from methodological adaptations that accommodate age differences. Future investigation could explore this distinction by employing identical, non‐age‐adapted targets across different developmental stages to determine whether specific eye‐tracking measures retain relevance independent of age‐based modifications. Such an approach could clarify whether refinement of eye‐tracking methodologies is necessary to improve sensitivity to developmental variations in attentional patterns.

Despite documented sex differences in eye‐tracking measures of social attention and gaze behaviors (Harrop et al. 2018, 2019; Kleberg et al. 2019; Putnam et al. 2023; Whyte and Scherf 2018), our work revealed no moderating effect of sex on pre–post treatment changes in eye‐tracking outcomes among autistic individuals. This finding contrasts with Mao et al. (2024) meta‐analytic results, which suggested that autistic males (70%) benefitted more from widely used autism interventions, such as applied behavior analysis and cognitive behavioral therapy, compared to only 45% of autistic females achieving comparable progress. A potential explanation lies in the composition of our sample, which, similar to much of the autism intervention literature, was predominantly male and encompassed various intervention types—ranging from behavioral to biomedical and technology‐based interventions—potentially diluting the sex differences observed in more traditional models (Mao et al. 2024). Consequently, our null result may not indicate a true absence of sex‐linked effects but could instead reflect limitations related to sample composition and heterogeneity of interventions, which obscure more nuanced differences.

The absence of moderation by treatment duration presents a nuanced scenario, contributing to the mixed evidence on this topic. Our result aligns with Sandbank et al. (2024) meta‐analytic finding, which investigated intervention amount (i.e., daily intensity, duration, and cumulative intensity) and found no consistent association with treatment effects in young autistic children. Conversely, Virués‐Ortega's (2010) dose–response analysis demonstrated that greater total hours of applied behavioral analytic intervention (intensity × duration) are associated with enhanced gains in language and adaptive skills among young autistic children. Additionally, Frazier et al. (2025), reanalyzing Sandbank's dataset, reported that intervention quantity predicts outcomes when controlling for child IQ. These conflicting findings may be partly attributable to differing analytic approaches, including variations in targeted interventions, dosage metrics, and the modeling of confounding variables, all of which could influence whether duration appears meaningful. It is noteworthy that in our study, duration was expressed in weeks of intervention. However, autism interventions vary greatly in intensity, structure, and fidelity; for instance, two interventions spanning 12 weeks may differ substantially in exposure, such as 1 h per week versus daily sessions. Therefore, simply reporting the calendar length may be an overly coarse measure, insufficient to capture the true dosage effects.

### 3.2 Eye Tracking as a Tool for Predicting Treatment Response

This work revealed a small‐to‐moderate, yet statistically nonsignificant, summary effect size concerning the correlation between baseline eye‐tracking outcomes and developmental change from pre‐ to post‐treatment among autistic individuals. Nonetheless, the identified moderators offer valuable insights.

Sex emerged as a significant moderator, such that a higher proportion of autistic male participants exhibited a weaker association between baseline eye‐tracking outcomes and subsequent changes in developmental domains. This finding aligns with a well‐established body of research indicating that autistic males and females often display distinct profiles across behavioral, neural, and genetic dimensions (e.g., Baron‐Cohen et al. 2005; Werling and Geschwind 2013). Notably, evidence suggests that autistic females often exhibit more normative patterns of social attention resembling typical development, whereas autistic males demonstrate more substantial nonsocial attentional biases and reduced social attention (Harrop et al. 2018, 2019; Putnam et al. 2023). These sex‐based differences may reflect underlying mechanistic variations. In autistic females, eye‐tracking measures may capture dynamic and adaptable social‐cognitive processes such as social interest, reward sensitivity, or attentional orienting, which are more amenable to intervention. Conversely, gaze behavior in autistic males may reflect more entrenched perceptual preferences or diminished sensitivity to social contingencies, thereby limiting the predictive utility of these metrics for relatively short‐term developmental change. Importantly, this sex moderation underscores the necessity for sex‐stratified approaches in biomarker development and intervention strategies. Rather than presuming that eye‐tracking measures possess universal predictive value across populations, future research may consider sex as a critical factor shaping their interpretive and predictive utility.

The developmental domain also significantly moderated the predictive association. Specifically, the association with cognitive changes—such as changes in visual reception and fine motor skills measured by the nonverbal developmental quotient (e.g., Bent et al. 2023)—was stronger compared to behavioral changes (e.g., adaptive functioning as measured by VABS), This suggests that visual attention functions as a foundational mechanism underpinning cognitive development in autism. For example, increased baseline attention to socially relevant stimuli could indicate readiness to engage in cognitive‐focused interventions, thereby enhancing these outcomes. However, it is important to recognize that many of the eye‐tracking targets at baseline were inherently social. This raises the possibility that the study design—anticipating a direct correlation between social attention and social outcomes—may not fully encapsulate the complexities of social development. Conversely, a weaker association between baseline attentional profiles and behavioral changes likely reflects the multifactorial nature of behavior, influenced by environmental factors such as caregiver involvement (Chung and Meadan 2021), and parental interaction styles (Patterson et al. 2014), as well as potential limitations in expecting social attention measures to predict long‐term social outcomes reliably. These broader influences may attenuate the direct impact of attentional profiles on behavioral changes, highlighting the need for complementary assessment tools to capture this complexity. Variability in observed associations may also be attributable to differences in the validity and measurement characteristics of cognitive and behavioral assessments. Cognitive assessments, such as MSEL, provide standardized, task‐based evaluations that are often more uniform across administrations. In contrast, behavioral assessments, such as ADOS, rely on clinical observations that may be more susceptible to contextual influences and variation in administration. These differences in construct validity and measurement approach likely contribute to heterogeneity in reported associations.

Importantly, the absence of moderation by publication year, participant age, and treatment duration aligns with our prior findings, which revealed no moderation of treatment‐related changes in eye‐tracking outcomes by these variables. While this consistency suggests a degree of stability in eye‐tracking outcomes across developmental stages and contexts, the absence of a significant summary effect size for the predictive association warrants cautious interpretation. Collectively, these results do not substantiate strong claims regarding the predictive utility of eye‐tracking measures for treatment outcomes. Instead, they emphasize the necessity for further investigation of the conditions under which eye‐tracking data may hold predictive value.

## 4 Limitations

It is important to acknowledge the limitations when interpreting and generalizing the results of this study. First, our methodology did not include backward or forward searches of references from the included studies; this omission could have excluded potentially relevant studies that might have provided valuable insights. Second, although 39 effect sizes were used to analyze baseline eye‐tracking profiles as predictive markers of treatment outcomes for autistic individuals, these effect sizes were derived from a small number of studies; this limited sample may restrict the robustness and generalizability of our findings, and therefore, the analysis was positioned as exploratory. Third, it is important to recognize the limitations of using correlation coefficients as predictive markers. Correlation measures the linear association between baseline eye‐tracking profiles and developmental change but does not imply causality. For example, an autistic individual's baseline attentional profile might predict treatment gain because gaze facilitates learning, as they are influenced by a third factor, such as underlying neural connectivity and language exposure, or due to coincidental measurement overlaps. Correlations could also mask heterogeneity in individual trajectories. For example, a small‐to‐moderate correlation at the group level might mask subgroups where gaze is highly predictive and others where it is not relevant. Relying solely on correlation risks oversimplifying this heterogeneity to an average that may not translate well to individual clinical prediction. Moreover, correlations from published studies could be affected by factors such as range restriction and measurement artifacts, which can distort estimates of predictive power. Fourth, grouping treatment types relatively broadly was necessary to evaluate their role as moderators. For example, we classified a study targeting speech as ‘behavioral treatment’ and one using transcranial magnetic stimulation as ‘biomedical treatment.’ While the targeted construct had homogeneity within each classified type, this grouping may have oversimplified the complexities of treatment and introduced biases that could influence the results. The statistical power to detect the moderator effect for treatment type may also have been insufficient. Finally, other potential moderators have limitations. Variables that could additionally influence the associated effect sizes were not fully examined due to constraints such as limited data reporting, lack of variability among some variables, and inconsistencies that made grouping into specific categories unfeasible. Additionally, all moderators were assessed separately, which presents a limitation. This univariate approach could have masked potential interactions among moderators, such as between treatment type and duration. Due to the number of categorical levels and the limited available effect sizes, modeling interactions would have increased the complexity of the model and reduced the reliability of the estimates. Similarly, although we recognize differences in validity between behavioral measures (e.g., ADOS) and cognitive measures (e.g., MSEL), subgroup analyses based on these measurement tools were not feasible due to the small number of studies. As a result, the moderators examined may not fully capture all factors influencing the observed effect sizes, and analyzing them separately may have obscured potential interactions.

## 5 Implications

The implications of the findings underscore the evolving role of eye tracking in autism research and intervention, particularly in their application for understanding both the short‐term and long‐term impacts of autism treatments. Our findings suggest that eye‐tracking outcomes in autistic individuals can change with intervention; however, these changes are most evident in contexts where psychosocial cues are highly salient, under treatments that directly affect neurobiological systems, and when measured with dynamic stimuli. Although the effect size is small to moderate—and when considered against MCIDs from related autism outcomes, suggests these are genuine, measurable changes—these changes are primarily incremental and unlikely to alter social functioning independently. For research and practice, this implies that eye‐tracking measures can serve as a valuable mechanistic indicator of treatment impact; nonetheless, it should be interpreted alongside measures of functional change and embedded within trial design that clarify whether changes in eye‐tracking outcomes meaningfully translate into the lived experiences of autistic individuals and their families. Conversely, our findings suggest that the predictive utility of baseline eye‐tracking profiles offers a detectable signal, though one that is conditional and domain specific. The moderating effects of sex and developmental domain suggest that this predictive utility is neither uniform nor straightforward; it is likely more robust for cognitive outcomes and groups with greater attentional flexibility (e.g., autistic females), while weaker for predicting behaviorally complex outcomes. This distinction highlights an important limitation in the current evidence base; utilizing baseline eye‐tracking profiles as predictors of treatment response remains preliminary. Future research may build on this insight by investigating whether post‐treatment changes in eye‐tracking outcomes correlate with changes in developmental outcomes, providing a more direct and clinically relevant framework for appraising the predictive value of eye‐tracking profiles and intervention efficacy. Improved study designs—incorporating longitudinal tracking and more granular behavioral and cognitive outcome measures—are essential to elucidate the role of eye tracking in monitoring and predicting developmental change in response to treatment. Collectively, these implications advocate for a dual approach in future research: refining the application of eye tracking as a sensitive and reliable tool for monitoring treatment effects, while also developing more targeted methods to optimize its predictive capacity across various developmental domains. Such efforts could bridge the gap between short‐term intervention outcomes and long‐term developmental trajectories, ultimately advancing the goal of individualized and adaptive autism treatment.

## 6 Conclusion

Our findings reveal the potential for eye tracking as a tool for monitoring treatment‐related changes in autism, while also emphasizing current limitations in its predictive utility. Moving forward, it will be essential to enhance both methodological precision and conceptual integration of eye‐tracking data within broader developmental frameworks. Such advancements can facilitate the development of more personalized and adaptive interventions and deepen our understanding of how gaze and attentional patterns contribute to change among autistic individuals.

## Author Contributions

C.D.Y., Y.X., A.K.T., H.M., and F.S. conceptualized the study. C.D.Y. and A.K.T. evaluated the data. C.D.Y. and Y.X. conducted the analyses. C.D.Y. drafted the manuscript. All authors (C.D.Y., Y.X., A.K.T., H.M., F.S.) revised the manuscript and approved the final version.

## Ethics Statement

The authors have nothing to report.

## Conflicts of Interest

Frederick Shic is or has been a consultant for MindMed Inc., Janssen Pharmaceuticals, and Roche Ltd. The other authors declare no conflicts of interest.

## Supporting information


**Figure S1:** Cook's distance.
**Figure S2:** Distribution of effect sizes for changes in eye‐tracking outcomes from pre‐ to post‐treatment in autistic individuals.
**Figure S3:** Distribution of effect sizes for the correlation between baseline (i.e., pre‐treatment) eye‐tracking outcomes and changes in developmental outcomes from pre‐ to post‐treatment in autistic individuals.

**Table S1:** An explanation of moderator selection.

**Table S2:** Quality assessment of included studies with effect sizes associated with changes in eye‐tracking outcomes from pre‐ to post‐treatment (*n* = 21).
**Table S3:** Quality assessment of included studies with effect sizes associated with the correlation between baseline (i.e., pre‐treatment) eye‐tracking outcomes and changes in developmental outcomes from pre‐ to post‐treatment (*n* = 4).

**Table S4:** Characteristics of included studies with effect sizes associated with changes in eye‐tracking outcomes from pre‐ to post‐treatment (*k* = 179 effect sizes).
**Table S5:** Characteristics of included studies with effect sizes associated with correlation between baseline (i.e., pre‐treatment) eye‐tracking outcomes and changes in developmental outcomes from pre‐ to post‐treatment (*k* = 39 effect sizes).

## Data Availability

Data sharing not applicable to this article as no datasets were generated or analyzed during the current study.
