# Supplementary material for: Eye Tracking as a Treatment Monitoring Tool for Autism: A Multilevel Meta‐Analysis
Source: Autism Res. 2025 Nov 14;18(12):2548–65. doi: 10.1002/aur.70141 (PMC12729504; doi:10.1002/aur.70141)

**SUPPLEMENTARY FIGURE 1** Cook’s distance.

1. Cook’s distance for changes in eye-tracking outcomes from pre- to post-treatment


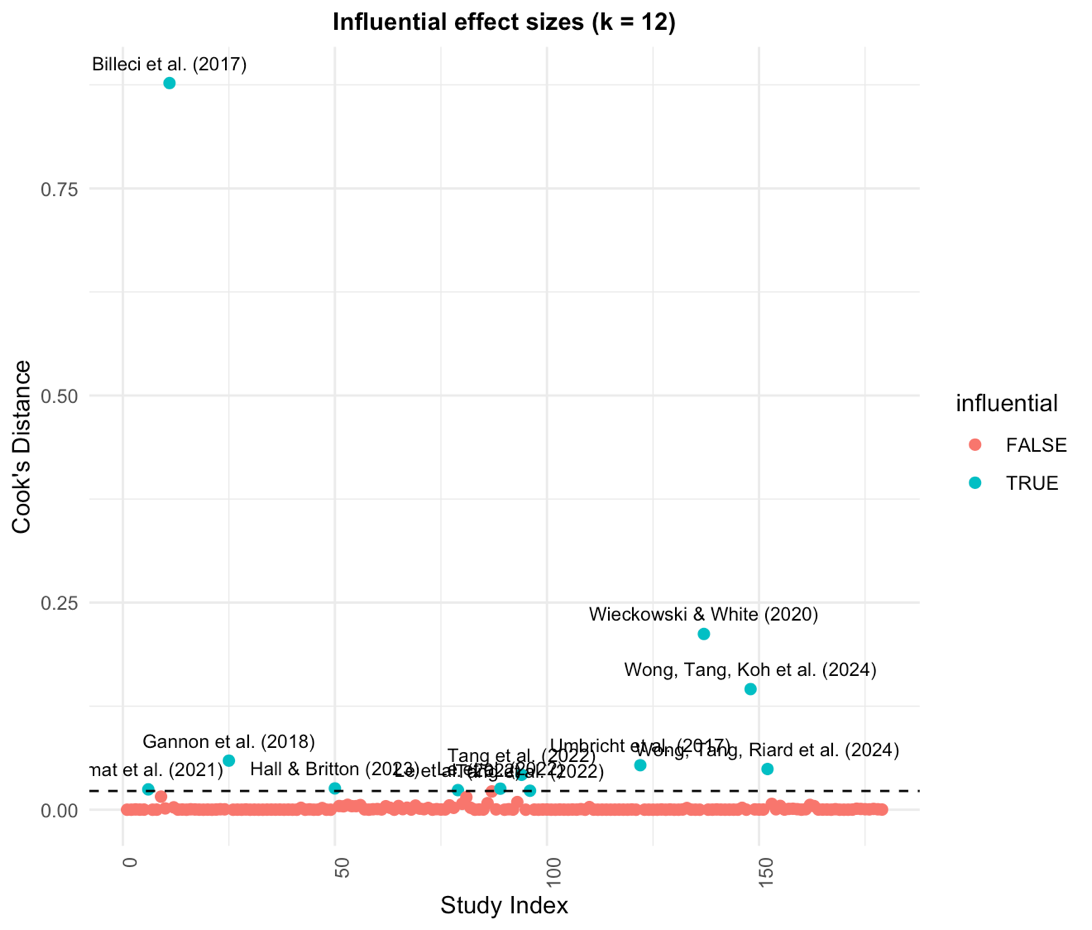


1. Cook’s distance for the correlation between baseline (pre-treatment) eye-tracking outcomes and changes in developmental outcomes from pre- to post-treatment


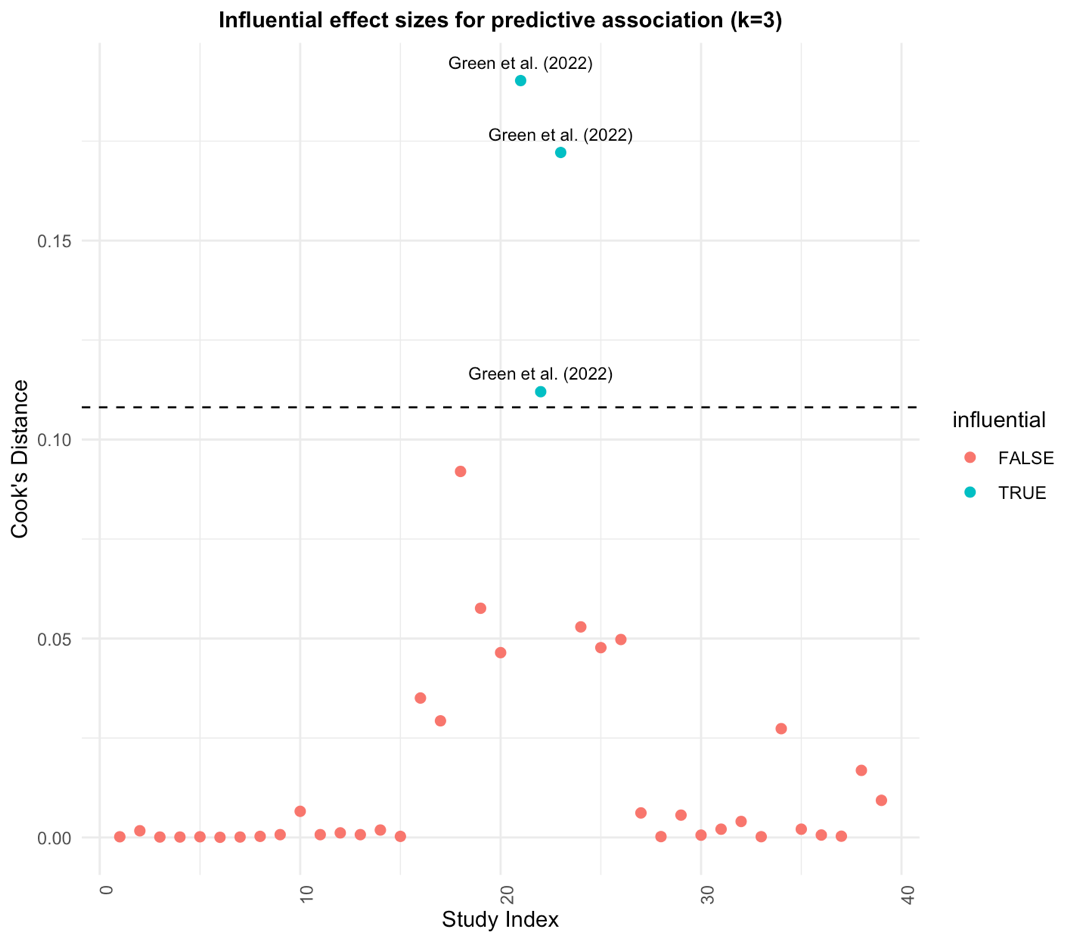


**SUPPLEMENTARY FIGURE 2** Distribution of effect sizes for changes in eye-tracking outcomes from pre- to post-treatment in autistic individuals.

1. Before removing outliers (*k* = 179)


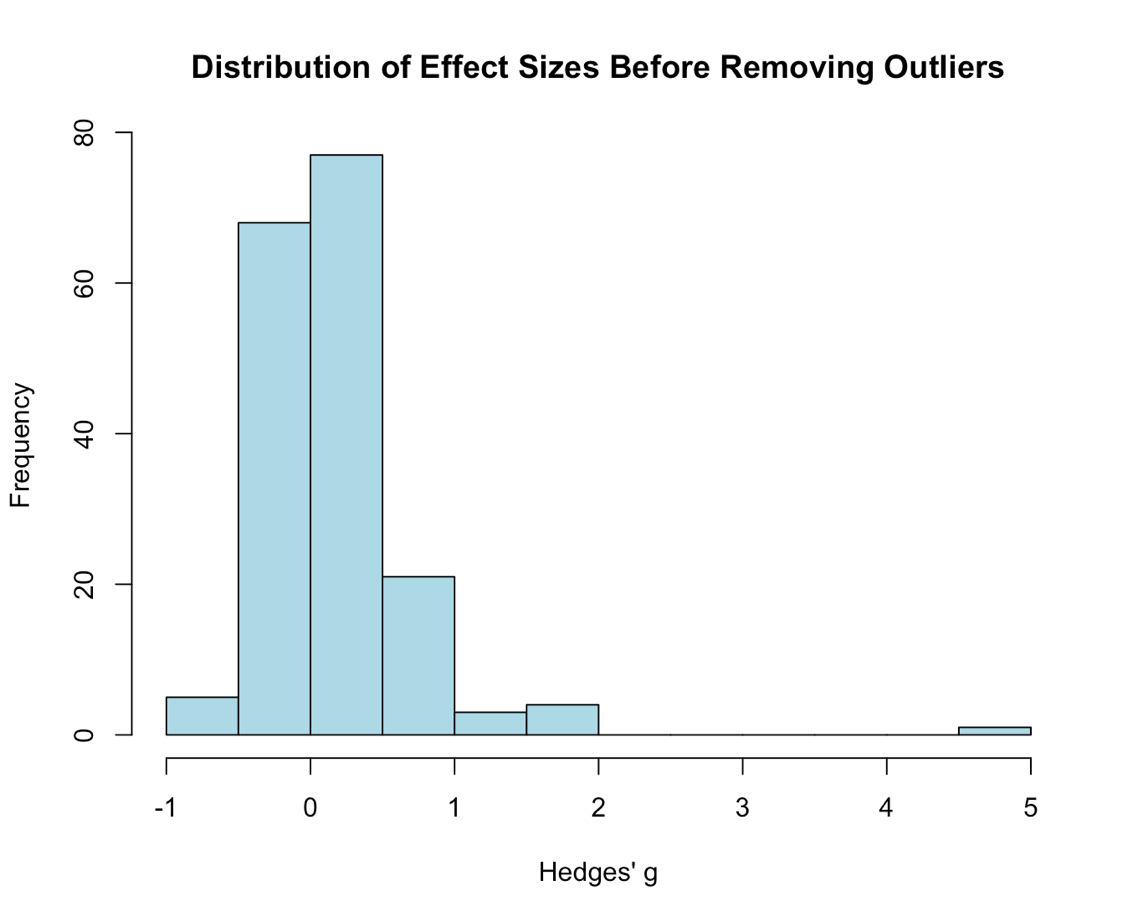


1. After removing outliers (*k* = 167)


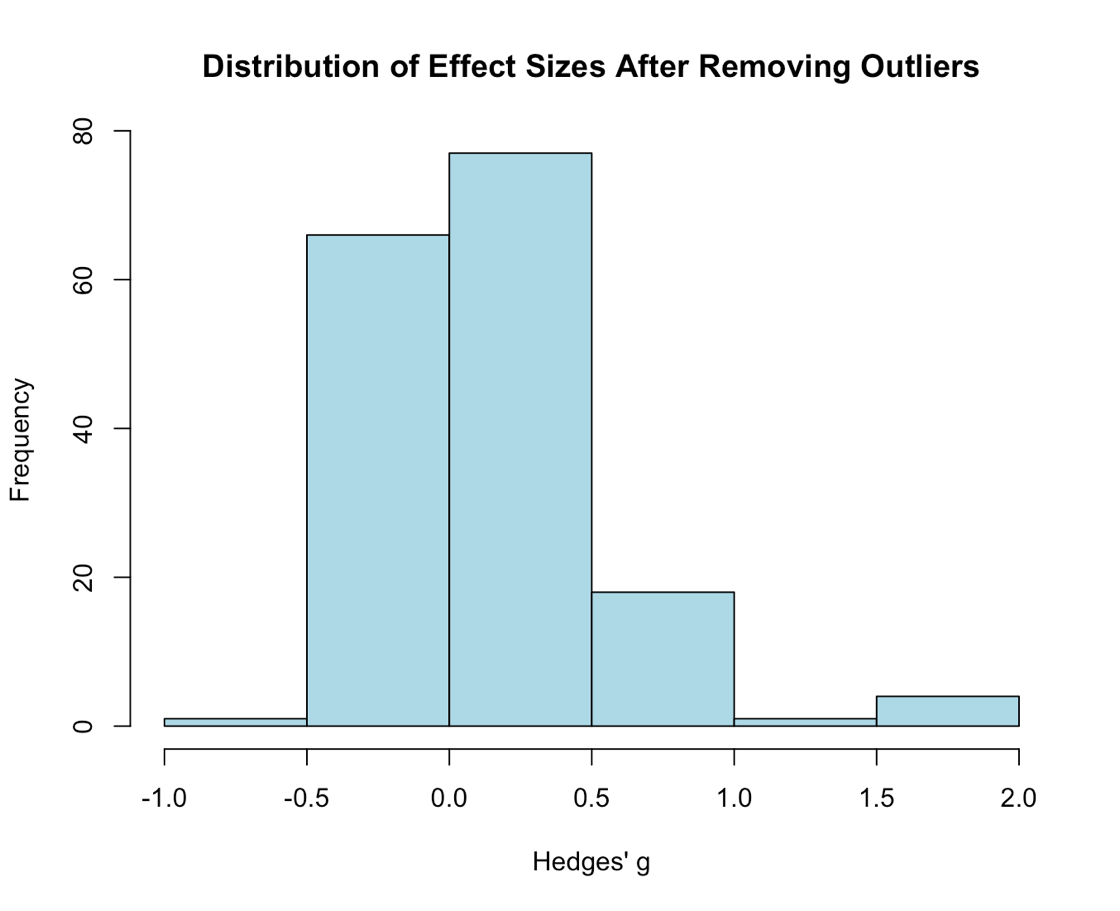


**SUPPLEMENTARY FIGURE 3** Distribution of effect sizes for the correlation between baseline (i.e., pre-treatment) eye-tracking outcomes and changes in developmental outcomes from pre- to post-treatment in autistic individuals.

1. Before removing outliers (*k* = 39)


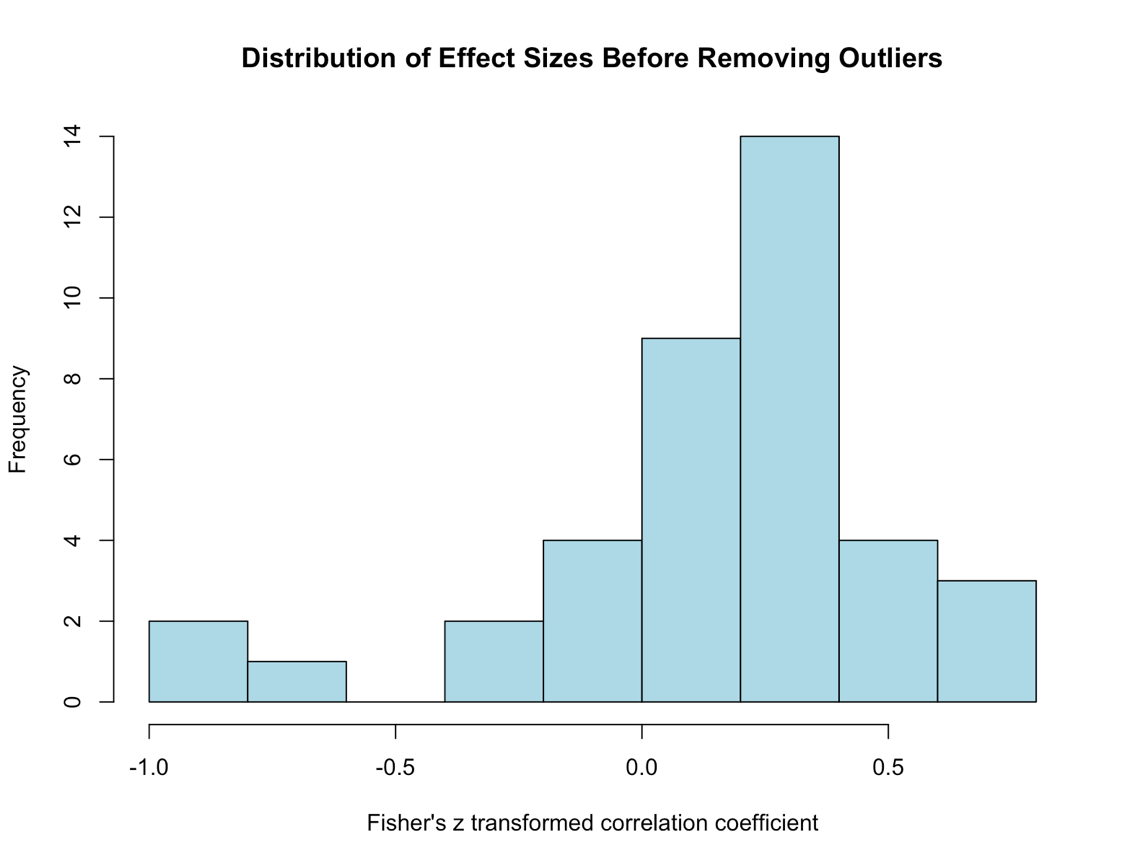


1. After removing outliers (*k* = 36)


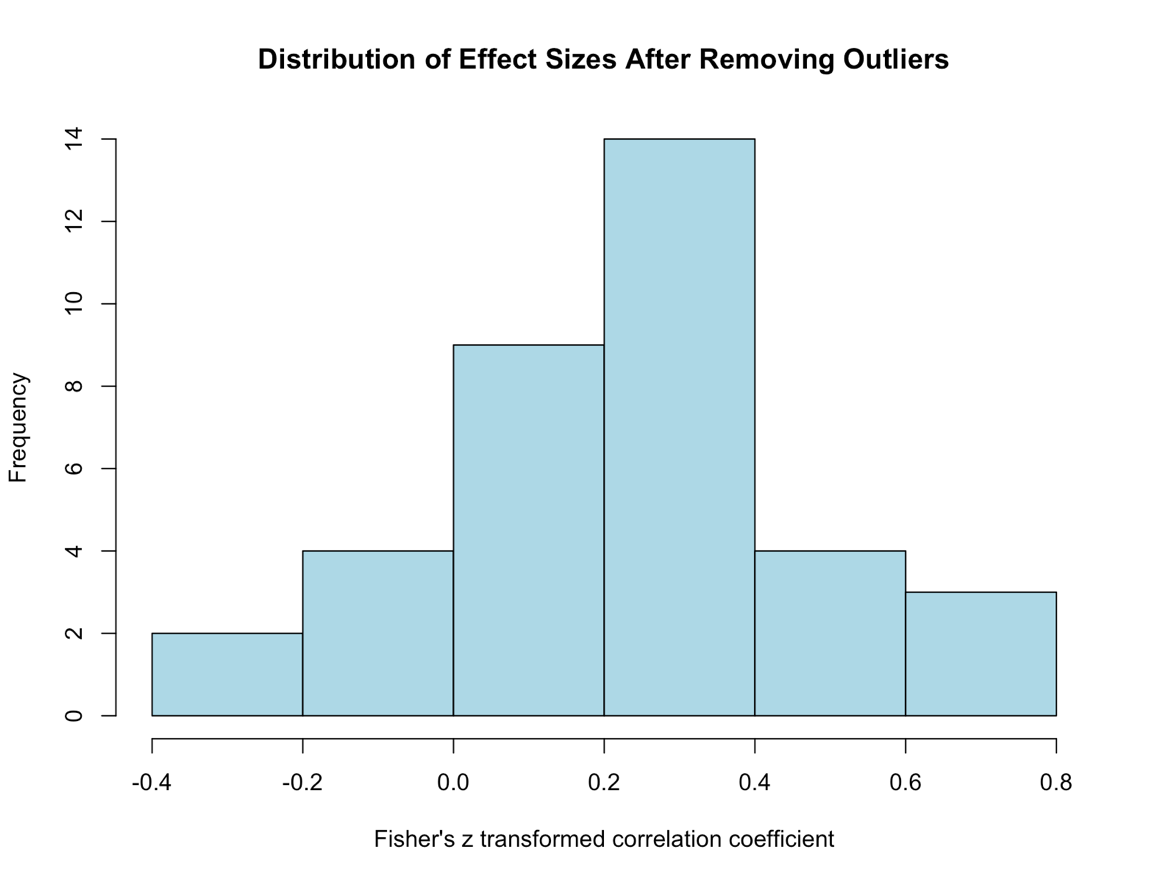

Supplement: Supplementary file 1 — Figure S1: Cook's distance. Figure S2: Distribution of effect sizes for changes in eye‐tracking outcomes from pre‐ to post‐treatment in autistic individuals. Figure S3: Distribution of effect sizes for the correlation between baseline (i.e., pre‐treatment) eye‐tracking outcomes and changes in developmental outcomes from pre‐ to post‐treatment in autistic individuals. [file AUR-18-2548-s001.docx]
