# Supplementary material for: Eye Tracking as a Treatment Monitoring Tool for Autism: A Multilevel Meta‐Analysis
Source: Autism Res. 2025 Nov 14;18(12):2548–65. doi: 10.1002/aur.70141 (PMC12729504; doi:10.1002/aur.70141)
Supplement: Supplementary file 2 — Table S1: An explanation of moderator selection. [file AUR-18-2548-s003.docx]

**SUPPLEMENTARY TABLE 1** An explanation of moderator selection.

| **Moderator** | **Explanation** |
| --- | --- |
| Participant characteristics: age and sex | We evaluate characteristics of participants—age and sex—due to conflicting evidence in the literature. While some meta-analyses have shown no significant moderating effect of age on eye-tracking outcomes (Chita-Tegmark et al., 2016; Frazier et al., 2017; Riddiford et al., 2022), other studies suggest that age influences gaze behaviors in autistic individuals (Hao et al., 2018; Ma et al., 2021; Yoon et al., 2025), affecting how they attend to different facial regions. Clarifying this association is crucial for understanding developmental pathways, optimizing intervention strategies, and corresponding outcome measures to various age groups. Similarly, while one meta-analysis reported no significant effect of sex (Frazier et al., 2017), another highlighted the limitation of the predominance of male participants in eye-tracking studies in autism research (Riddiford et al., 2022), making firm conclusions difficult. Nevertheless, emerging evidence suggests distinct social attention profiles between sexes (Harrop et al., 2018; 2019; Kleberg et al., 2019; Putnam et al., 2023; Whyte & Scherf, 2018). As recognition of sex-specific autism presentations grows, accounting for sex differences in eye-tracking responses to treatment is essential for a deeper understanding of visual processing and social functioning in this population. |
| Study design: randomization and blinding status to treatment | We evaluate study design—specifically randomization and blinding—as both are instrumental in mitigating bias and ensuring that observed treatment outcomes are truly attributable to the treatment itself rather than confounding factors (Kasari, 2002; Sandbank et al., 2020). Randomization ensures that both known and unknown variables are evenly distributed, thereby preventing selection bias. Blinding minimizes the risk of expectation and observer bias by maintaining treatment conditions concealed from participants and researchers. Therefore, assessing these factors can help determine whether observed changes in eye-tracking outcomes following treatment are accurate or biased. |
| Treatment approach: type and duration | We evaluate whether different treatment types and their durations influence changes in eye-tracking outcomes. Given the heterogeneity of autism interventions—from behavioral therapies to pharmacological and technology-based interventions (Kaye et al., 2024)—and evidence that treatment duration can differentially impact developmental domains (Linstead et al., 2017), examining how treatment type and duration influence attentional patterns could provide insights into mechanisms of change and inform the development of more tailored, effective interventions. |
| Developmental domain | We evaluate whether the targeted developmental domain moderates the predictive association between baseline eye-tracking profiles and developmental changes following treatment, as prior research suggests that interventions may differentially impact specific domains (e.g., cognitive, language, behavioral outcomes; Linstead et al., 2017; Shi et al., 2021). This evaluation will help determine whether baseline eye-tracking profiles hold domain-specific predictive value. |
| Eye-tracking stimulus motion format | We evaluate the motion format of eye-tracking stimuli (dynamic, static) due to evidence suggesting that the naturalness spectrum of stimuli may influence eye-tracking outcomes (Yoon et al., 2025). Additionally, prior studies suggest that motion format impacts how gaze behaviors differ between autistic individuals and controls, with dynamic stimuli often eliciting more pronounced group differences (Chevallier et al., 2015; Cilia et al., 2019; Saitovitch et al., 2013; Speer et al., 2007). Understanding whether motion format similarly moderates treatment-related changes in eye-tracking outcomes could inform future experimental designs and enhance the ecological validity of eye-tracking research in autism. |
| Publication year | We evaluate publication year to determine whether effect sizes in eye-tracking studies have evolved. This could reflect methodological advancements in eye tracking, evolving diagnostic criteria, or shifts in intervention strategies. Previous meta-analysis findings observed a trend of decreasing effect sizes for differences between individuals with and without autism in more recent publications (Frazier et al., 2017). This suggests that examining publication years may reveal a similar trend of improvements in methodological rigor and reporting quality over time in eye-tracking studies related to treatment outcomes. |

**References**

Chevallier, C., Parish-Morris, J., McVey, A., Rump, K. M., Sasson, N. J., Herrington, J. D., & Schultz, R. T. (2015). Measuring social attention and motivation in autism spectrum disorder using eye-tracking: Stimulus type matters. *Autism Research*, *8*(5), 620–628. <https://doi.org/10.1002/aur.1479>

Chita-Tegmark, M. (2016). Social attention in ASD: A review and meta-analysis of eye-tracking studies.

*Research in Developmental Disabilities*, *48*, 79–93.<https://doi.org/10.1016/j.ridd.2015.10.011>

Cilia, F., Aubry, A., Le Driant, B., Bourdin, B., & Vandromme, L. (2019). Visual exploration of dynamic or static joint attention bids in children with autism syndrome disorder. *Frontiers in Psychology*, *10*, 2187. <https://doi.org/10.3389/fpsyg.2019.02187>

Frazier, T. W., Strauss, M., Klingemier, E. W., Zetzer, E. E., Hardan, A. Y., Eng, C., & Youngstrom, E. A.

(2017). A meta-analysis of gaze differences to social and nonsocial information between individuals

with and without autism. *Journal of the American Academy of Child & Adolescent Psychiatry*, *56*(7),

546–555. <https://doi.org/10.1016/j.jaac.2017.05.005>

Hao, Y., Wang, F., Xie, H., An, J., Wang, Y., & Liu, H. (2018). How do autism spectrum disorders process

human face? A meta-analysis of eye-tracking studies. *Advances in Psychological Science*, *26*(1), 26.

https://doi.org/[10.3724/SP.J.1042.2018.00026](https://doi.org/10.3724/SP.J.1042.2018.00026)

Harrop, C., Jones, D., Zheng, S., Nowell, S., Schultz, R., & Parish-Morris, J. (2019). Visual attention to faces in children with autism spectrum disorder: Are there sex differences? *Molecular Autism*, *10*(1), 28. https://doi.org/10.1186/s13229-019-0276-2

Harrop, C., Jones, D., Zheng, S., Nowell, S. W., Boyd, B. A., & Sasson, N. (2018). Sex differences in social attention in autism spectrum disorder. *Autism Research*, *11*(9), 1264–1275. <https://doi.org/10.1002/aur.1997>

Kasari, C. (2002). Assessing change in early intervention programs for children with autism. *Journal of Autism*

*and Developmental Disorders*, *32*(5), 447-461. <https://doi.org/10.1023/A:1020546006971>

Kaye, A. D., Allen, K. E., Smith III, V. S., Tong, V. T., Mire, V. E., Nguyen, H., Lee, Z., Kouri, M., Jean

Baptiste, C., Mosieri, C. N., Kaye, A. M., Varrassi, G., & Shekoohi, S. (2024). Emerging treatments and

therapies for autism spectrum disorder: A narrative review. *Cureus, 16*(7), e63671. <https://doi.org/10.7759/cureus.63671>

Kleberg, J. L., Nyström, P., Bölte, S., & Falck-Ytter, T. (2019). Sex differences in social attention in infants at

risk for autism. *Journal of Autism and Developmental Disorders*, *49*(4), 1342–1351. <https://doi.org/10.1007/s10803-018-3799-z>

Linstead, E., Dixon, D. R., Hong, E., Burns, C. O., French, R., Novack, M. N., & Granpeesheh, D. (2017). An

evaluation of the effects of intensity and duration on outcomes across treatment domains for children

with autism spectrum disorder. *Translational Psychiatry*, *7*(9), e1234–e1234. <https://doi.org/10.1038/tp.2017.207>

Ma, X., Gu, H., & Zhao, J. (2021). Atypical gaze patterns to facial feature areas in autism spectrum disorders

reveal age and culture effects: A meta‐analysis of eye‐tracking studies. *Autism Research*, *14*(12), 2625–

2639.<https://doi.org/10.1002/aur.2607>

Putnam, O. C., Sasson, N., Parish‐Morris, J., & Harrop, C. (2023). Effects of social complexity and gender on social and non‐social attention in male and female autistic children: A comparison of four eye‐tracking paradigms. *Autism Research*, *16*(2), 315–326. <https://doi.org/10.1002/aur.2851>

Riddiford, J. A., Enticott, P. G., Lavale, A., & Gurvich, C. (2022). Gaze and social functioning associations in

autism spectrum disorder: A systematic review and meta-analysis. *Autism Research*, *15*(8), 1380–1446. <https://doi.org/10.1002/aur.2729>

Saitovitch, A., Bargiacchi, A., Chabane, N., Phillipe, A., Brunelle, F., Boddaert, N., Samson, Y., & Zilbovicius, M. (2013). Studying gaze abnormalities in autism: Which type of stimulus to use? *Open Journal of Psychiatry*, *3*(2), 32–38. <https://doi.org/10.4236/ojpsych.2013.32A006>

Sandbank, M., Bottema-Beutel, K., Crowley, S., Cassidy, M., Dunham, K., Feldman, J. I., Crank, J., Albarran, S. A., Raj, S., Mahbub, P., & Woynaroski, T. G. (2020). Project AIM: Autism intervention meta-analysis for studies of young children. *Psychological Bulletin*, *146*(1), 1–29. <https://doi.org/10.1037/bul0000215>

Shi, B., Wu, W., Dai, M., Zeng, J., Luo, J., Cai, L., Wan, B., & Jing, J. (2021). Cognitive, language, and

behavioral outcomes in children with autism spectrum disorders exposed to early comprehensive

treatment models: A meta-analysis and meta-regression. *Frontiers in Psychiatry*, *12*, 691148. <https://doi.org/10.3389/fpsyt.2021.691148>

Speer, L. L., Cook, A. E., McMahon, W. M., & Clark, E. (2007). Face processing in children with autism: Effects of stimulus contents and type. *Autism*, *11*(3), 265–277. <https://doi.org/10.1177/1362361307076925>

Whyte, E. M., & Scherf, K. S. (2018). Gaze following is related to the broader autism phenotype in a sex-

specific way: Building the case for distinct male and female autism phenotypes. *Clinical Psychological*

*Science*, *6*(2), 280–287. <https://doi.org/10.1177/2167702617738380>

Yoon, C. D., Xia, Y., Terol, A. K., Meadan, H., & Lee, J. D. (2025). Correlation between gaze behaviors and

social communication skills of young autistic children: A meta-analysis of eye-tracking studies. *Journal*

*of Autism and Developmental Disorders, 55*, 843-861. <https://doi.org/10.1007/s10803-024-06257-x>
