# Supplementary material for: Eye Tracking as a Treatment Monitoring Tool for Autism: A Multilevel Meta‐Analysis
Source: Autism Res. 2025 Nov 14;18(12):2548–65. doi: 10.1002/aur.70141 (PMC12729504; doi:10.1002/aur.70141)
Supplement: Supplementary file 3 — Table S2: Quality assessment of included studies with effect sizes associated with changes in eye‐tracking outcomes from pre‐ to post‐treatment (n = 21). Table S3: Quality assessment of included studies with effect sizes associated with the correlation between baseline (i.e., pre‐treatment) eye‐tracking outcomes and changes in developmental outcomes from pre‐ to post‐treatment (n = 4). [file AUR-18-2548-s002.pdf]

SUPPLEMENTARY TABLE 2 Quality assessment of included studies with effect sizes associated with changes in eye-tracking outcomes from pre- to post-treatment (*n* = 21).

**RoB 2.0 for randomized studies (*n* = 13)**						
Authors (year)	Randomization process	Deviations from intended interventions	Missing outcome data	Measurement of the outcome	Selection of the reported result	Overall judgment
Bradshaw et al. (2019)	some concerns	some concerns	some concerns	low risk	some concerns	some concerns
Dawson et al. (2020)	low risk	low risk	low risk	low risk	low risk	low risk
Gannon et al. (2018)	some concerns	some concerns	low risk	low risk	low risk	some concerns
Gepner et al. (2022)	some concerns	low risk	low risk	low risk	some concerns	some concerns
Hall & Britton (2023)	some concerns	some concerns	low risk	low risk	some concerns	some concerns
Le et al. (2022)	low risk	low risk	low risk	low risk	low risk	low risk
Scherf et al. (2024)	low risk	some concerns	low risk	low risk	low risk	some concerns
Schmitt et al. (2023)	low risk	low risk	low risk	low risk	low risk	low risk
Tang et al. (2022)	some concerns	low risk	low risk	low risk	low risk	some concerns
Umbricht et al. (2017)	low risk	low risk	low risk	low risk	low risk	low risk
Yamasue et al. (2020)	low risk	low risk	low risk	low risk	low risk	low risk
Zamzow et al. (2014)	low risk	low risk	some concerns	low risk	some concerns	some concerns
Zhao et al. (2024)	low risk	low risk	low risk	low risk	low risk	low risk

**ROBINS-I for non-randomized studies (*n* = 8)**							
Authors (year)	Confounding	Selection of participants	Classification of interventions	Deviations from intended interventions	Missing data	Measurement of outcomes	Selection of reported results	Overall judgment
Amaral et al. (2017)	moderate risk	low risk	low risk	low risk	low risk	low risk	low risk	moderate risk
Amat et al. (2021)	moderate risk	low risk	low risk	low risk	low risk	low risk	moderate risk	moderate risk
Billeci et al. (2017)	moderate risk	moderate risk	low risk	moderate risk	low risk	low risk	moderate risk	moderate risk
Dawson et al. (2017)	moderate risk	moderate risk	low risk	low risk	moderate risk	low risk	moderate risk	moderate risk
Tian et al. (2023)	moderate risk	low risk	low risk	low risk	low risk	low risk	moderate risk	moderate risk
Wieckowski & White (2020)	moderate risk	moderate risk	low risk	low risk	low risk	low risk	moderate risk	moderate risk
Wong, Tang, Koh et al. (2024)	moderate risk	moderate risk	low risk	low risk	low risk	low risk	low risk	moderate risk
Wong, Tang, Riard et al. (2024)	moderate risk	moderate risk	low risk	low risk	low risk	low risk	low risk	moderate risk

Note. RoB 2.0 is rated as low risk, some concerns, or high risk. ROBINS-I is rated as low risk, moderate risk, serious risk, or critical risk.

SUPPLEMENTARY TABLE 3 Quality assessment of included studies with effect sizes associated with the correlation between baseline (i.e., pre-treatment) eye-tracking outcomes and changes in developmental outcomes from pre- to post-treatment ($n = 4$)

**ROBINS-I for non-randomized studies**								
Authors (year)	Confounding	Selection of participants	Classification of interventions	Deviations from intended interventions	Missing data	Measurement of outcomes	Selection of reported results	Overall judgment
Bent et al. (2023)	low risk	moderate risk	low risk	moderate risk	low risk	moderate risk	moderate risk	moderate risk
Greene et al. (2021)	low risk	moderate risk	low risk	low risk	low risk	moderate risk	moderate risk	moderate risk
Robain et al. (2020)	low risk	moderate risk	low risk	moderate risk	low risk	moderate risk	moderate risk	moderate risk
Vivanti et al. (2013)	moderate risk	moderate risk	low risk	low risk	low risk	moderate risk	moderate risk	moderate risk
