# Supplementary material for: Eye Tracking as a Treatment Monitoring Tool for Autism: A Multilevel Meta‐Analysis
Source: Autism Res. 2025 Nov 14;18(12):2548–65. doi: 10.1002/aur.70141 (PMC12729504; doi:10.1002/aur.70141)
Supplement: Supplementary file 4 — Table S4: Characteristics of included studies with effect sizes associated with changes in eye‐tracking outcomes from pre‐ to post‐treatment (k = 179 effect sizes). Table S5: Characteristics of included studies with effect sizes associated with correlation between baseline (i.e., pre‐treatment) eye‐tracking outcomes and changes in developmental outcomes from pre‐ to post‐treatment (k = 39 effect sizes). [file AUR-18-2548-s004.docx]

**SUPPLEMENTARY TABLE 4** Characteristics of included studies with effect sizes associated with changes in eye-tracking outcomes from pre- to post-treatment (*k* = 179 effect sizes).

| **Authors (year)** | **Autistic Participants** | | |  | **Treatment** | | | | | |  | **Eye Tracking** | | ***g*** |
| --- | --- | --- | --- | --- | --- | --- | --- | --- | --- | --- | --- | --- | --- | --- |
|  | ***N*** | **mean age (years)** | **sex (%male)** |  | **randomization** | **blinding** | **dropout (%)** | **name** | **type** | **duration (weeks)** |  | **stimulus motion format** | **measure** |  |
| Amaral et al. (2018) | 15 | 22 | 100 |  | No | No | 0% | Brain-Computer Interface | technology | 17.38 |  | dynamic | fixation on target object of JA animation | 0.28 |
| Amaral et al. (2018) | 15 | 22 | 100 |  | No | No | 0% | Brain-Computer Interface | technology | 17.38 |  | dynamic | fixation on target object of JA animation preceded by fixation on avatar face | 0.30 |
| Amaral et al. (2018) | 15 | 22 | 100 |  | No | No | 0% | Brain-Computer Interface | technology | 17.38 |  | dynamic | fixation on target object of JA animation | 0.13 |
| Amaral et al. (2018) | 15 | 22 | 100 |  | No | No | 0% | Brain-Computer Interface | technology | 17.38 |  | dynamic | fixation on the target object of JA animation preceded by fixation on avatar face | 0.34 |
| Amat et al. (2021) | 9 | 11 | 56 |  | No | No | 0% | Interactive Virtual Reality System | technology | 2.86 |  | dynamic | highest point received when looked at the correct game object prompted by avatar | 0.60 |
| Amat et al. (2021) | 9 | 11 | 56 |  | No | No | 0% | Interactive Virtual Reality System | technology | 2.86 |  | dynamic | total time taken to interact with avatar and selecting game object for all prompts | 1.18 |
| Amat et al. (2021) | 9 | 11 | 56 |  | No | No | 0% | Interactive Virtual Reality System | technology | 2.86 |  | dynamic | time between when avatar provides a gaze prompt and when participant looks at the correct game object | 0.69 |
| Amat et al. (2021) | 9 | 11 | 56 |  | No | No | 0% | Interactive Virtual Reality System | technology | 2.86 |  | dynamic | total face fixation | 0.79 |
| Amat et al. (2021) | 9 | 11 | 56 |  | No | No | 0% | Interactive Virtual Reality System | technology | 2.86 |  | dynamic | normalized eye fixation | 0.24 |
| Amat et al. (2021) | 9 | 11 | 56 |  | No | No | 0% | Interactive Virtual Reality System | technology | 2.86 |  | dynamic | normalized other facial features fixation | 0.93 |
| Billeci et al. (2017) | 7 | 7.5 | 100 |  | No | No | 0% | Rehabilitative intervention on JA and imitation | behavioral | 26.07 |  | dynamic | cumulative fixation duration on face for control | 4.87 |
| Billeci et al. (2017) | 7 | 7.5 | 100 |  | No | No | 0% | Rehabilitative intervention on JA and imitation | behavioral | 26.07 |  | dynamic | cumulative fixation duration on face for RJA | 1.38 |
| Billeci et al. (2017) | 7 | 7.5 | 100 |  | No | No | 0% | Rehabilitative intervention on JA and imitation | behavioral | 26.07 |  | dynamic | cumulative fixation duration on face for IJA | 1.79 |
| Bradshaw et al. (2019) | 12 | 2.9 | 92 |  | Yes | No | 13% | Pivotal Response Intervention for Social Motivation | behavioral | 26.07 |  | static | mouth-eyes ratio | -0.02 |
| Bradshaw et al. (2019) | 11 | 2.9 | 82 |  | Yes | No | 8% | Assortment of EIs (ABA, speech, special needs preschool) | behavioral | 26.07 |  | static | mouth-eyes ratio | 0.06 |
| Bradshaw et al. (2019) | 12 | 2.9 | 92 |  | Yes | No | 13% | Pivotal Response Intervention for Social Motivation | behavioral | 26.07 |  | dynamic | social preference | 0.24 |
| Bradshaw et al. (2019) | 11 | 2.9 | 82 |  | Yes | No | 8% | Assortment of EIs (ABA, speech, special needs preschool) | behavioral | 26.07 |  | dynamic | social preference | -0.05 |
| Dawson et al. (2017) | 21 | 4.6 | 84 |  | No | No | 12% | autologous umbilical cord blood infusion | biomedical | 0.14 |  | dynamic | attention on eyes | 0.10 |
| Dawson et al. (2017) | 21 | 4.6 | 84 |  | No | No | 12% | autologous umbilical cord blood infusion | biomedical | 0.14 |  | dynamic | attention on actress | 0.01 |
| Dawson et al. (2017) | 21 | 4.6 | 84 |  | No | No | 12% | autologous umbilical cord blood infusion | biomedical | 0.14 |  | dynamic | attention on mouth | -0.04 |
| Dawson et al. (2017) | 21 | 4.6 | 84 |  | No | No | 12% | autologous umbilical cord blood infusion | biomedical | 0.14 |  | dynamic | attention on face | 0.01 |
| Dawson et al. (2020) | 176 | 5.4 | 79 |  | Yes | Yes | 2% | intravenous umbilical cord blood infusion | biomedical | 0.14 |  | dynamic | mean look duration during actress with dyadic bid | 1.86 |
| Dawson et al. (2020) | 176 | 5.4 | 79 |  | Yes | Yes | 2% | intravenous umbilical cord blood infusion | biomedical | 0.14 |  | dynamic | mean look duration during actress with moving toys | 1.90 |
| Dawson et al. (2020) | 176 | 5.4 | 79 |  | Yes | Yes | 2% | intravenous umbilical cord blood infusion | biomedical | 0.14 |  | dynamic | average look duration across actress with dyadic bid and with moving toys | 1.89 |
| Gannon et al. (2018) | 20 | 12.9 | 100 |  | Yes | No | 0% | behavioral skills training package | behavioral | 0.43 |  | live | % time engaged in social gaze with the examiner | 1.00 |
| Gepner et al. (2022) | 12 | 5.8 | 87 |  | Yes | Yes | 0% | speech therapy using Logiral | behavioral | 52.14 |  | dynamic | number of fixations on mouth | -0.08 |
| Gepner et al. (2022) | 12 | 5.8 | 87 |  | Yes | Yes | 0% | speech therapy using Logiral | behavioral | 52.14 |  | dynamic | number of fixations on face | -0.42 |
| Gepner et al. (2022) | 12 | 5.8 | 87 |  | Yes | Yes | 0% | speech therapy using Logiral | behavioral | 52.14 |  | dynamic | number of fixations on eyes | -0.36 |
| Gepner et al. (2022) | 12 | 5.8 | 87 |  | Yes | Yes | 0% | speech therapy using Logiral | behavioral | 52.14 |  | dynamic | number of fixations on outside | 0.06 |
| Gepner et al. (2022) | 12 | 5.8 | 87 |  | Yes | Yes | 0% | speech therapy using Logiral | behavioral | 52.14 |  | dynamic | mean fixation duration on mouth | -0.12 |
| Gepner et al. (2022) | 12 | 5.8 | 87 |  | Yes | Yes | 0% | speech therapy using Logiral | behavioral | 52.14 |  | dynamic | mean fixation duration on face | -0.30 |
| Gepner et al. (2022) | 12 | 5.8 | 87 |  | Yes | Yes | 0% | speech therapy using Logiral | behavioral | 52.14 |  | dynamic | mean fixation duration on eyes | -0.12 |
| Gepner et al. (2022) | 12 | 5.8 | 87 |  | Yes | Yes | 0% | speech therapy using Logiral | behavioral | 52.14 |  | dynamic | mean fixation duration on outside | -0.06 |
| Gepner et al. (2022) | 12 | 5.8 | 87 |  | Yes | Yes | 0% | speech therapy using Logiral | behavioral | 52.14 |  | dynamic | total fixation time on mouth | -0.25 |
| Gepner et al. (2022) | 12 | 5.8 | 87 |  | Yes | Yes | 0% | speech therapy using Logiral | behavioral | 52.14 |  | dynamic | total fixation time on face | -0.42 |
| Gepner et al. (2022) | 12 | 5.8 | 87 |  | Yes | Yes | 0% | speech therapy using Logiral | behavioral | 52.14 |  | dynamic | total fixation time on eyes | -0.25 |
| Gepner et al. (2022) | 12 | 5.8 | 87 |  | Yes | Yes | 0% | speech therapy using Logiral | behavioral | 52.14 |  | dynamic | total fixation time on outside | 0.01 |
| Gepner et al. (2022) | 11 | 5.8 | 87 |  | Yes | Yes | 0% | speech therapy without Logiral | behavioral | 52.14 |  | dynamic | number of fixations on mouth | 0.01 |
| Gepner et al. (2022) | 11 | 5.8 | 87 |  | Yes | Yes | 0% | speech therapy without Logiral | behavioral | 52.14 |  | dynamic | number of fixations on face | -0.26 |
| Gepner et al. (2022) | 11 | 5.8 | 87 |  | Yes | Yes | 0% | speech therapy without Logiral | behavioral | 52.14 |  | dynamic | number of fixations on eyes | 0.05 |
| Gepner et al. (2022) | 11 | 5.8 | 87 |  | Yes | Yes | 0% | speech therapy without Logiral | behavioral | 52.14 |  | dynamic | number of fixations on outside | -0.05 |
| Gepner et al. (2022) | 11 | 5.8 | 87 |  | Yes | Yes | 0% | speech therapy without Logiral | behavioral | 52.14 |  | dynamic | mean fixation duration on mouth | 0.36 |
| Gepner et al. (2022) | 11 | 5.8 | 87 |  | Yes | Yes | 0% | speech therapy without Logiral | behavioral | 52.14 |  | dynamic | mean fixation duration on face | -0.17 |
| Gepner et al. (2022) | 11 | 5.8 | 87 |  | Yes | Yes | 0% | speech therapy without Logiral | behavioral | 52.14 |  | dynamic | mean fixation duration on eyes | 0.23 |
| Gepner et al. (2022) | 11 | 5.8 | 87 |  | Yes | Yes | 0% | speech therapy without Logiral | behavioral | 52.14 |  | dynamic | mean fixation duration on outside | -0.06 |
| Gepner et al. (2022) | 11 | 5.8 | 87 |  | Yes | Yes | 0% | speech therapy without Logiral | behavioral | 52.14 |  | dynamic | total fixation time on mouth | 0.12 |
| Gepner et al. (2022) | 11 | 5.8 | 87 |  | Yes | Yes | 0% | speech therapy without Logiral | behavioral | 52.14 |  | dynamic | total fixation time on face | -0.41 |
| Gepner et al. (2022) | 11 | 5.8 | 87 |  | Yes | Yes | 0% | speech therapy without Logiral | behavioral | 52.14 |  | dynamic | total fixation time on eyes | 0.03 |
| Gepner et al. (2022) | 11 | 5.8 | 87 |  | Yes | Yes | 0% | speech therapy without Logiral | behavioral | 52.14 |  | dynamic | total fixation time on outside | -0.13 |
| Hall & Britton (2023) | 26 | 12.8 | 100 |  | Yes | No | 0% | behavioral treatment probe | behavioral | 0.29 |  | live | % mean dwell time on face | -0.19 |
| Le et al. (2022) | 18 | 5 | NA |  | Yes | Yes | 5% | intranasal oxytocin | biomedical | 6 |  | dynamic | % total fixation duration for dynamic social | 0.65 |
| Le et al. (2022) | 18 | 5 | NA |  | Yes | Yes | 5% | intranasal oxytocin | biomedical | 6 |  | static | % eyes-angry face | 0.72 |
| Le et al. (2022) | 18 | 5 | NA |  | Yes | Yes | 5% | intranasal oxytocin | biomedical | 6 |  | static | % eyes-fearful face | -0.39 |
| Le et al. (2022) | 18 | 5 | NA |  | Yes | Yes | 5% | intranasal oxytocin | biomedical | 6 |  | static | % eyes-happy face | 0.70 |
| Le et al. (2022) | 18 | 5 | NA |  | Yes | Yes | 5% | intranasal oxytocin | biomedical | 6 |  | static | % eyes-neutral face | 0.56 |
| Le et al. (2022) | 18 | 5 | NA |  | Yes | Yes | 5% | intranasal oxytocin | biomedical | 6 |  | static | % nose-angry face | -0.35 |
| Le et al. (2022) | 18 | 5 | NA |  | Yes | Yes | 5% | intranasal oxytocin | biomedical | 6 |  | static | % nose-fearful face | 0.20 |
| Le et al. (2022) | 18 | 5 | NA |  | Yes | Yes | 5% | intranasal oxytocin | biomedical | 6 |  | static | % nose-happy face | 0.07 |
| Le et al. (2022) | 18 | 5 | NA |  | Yes | Yes | 5% | intranasal oxytocin | biomedical | 6 |  | static | % nose-neutral face | -0.01 |
| Le et al. (2022) | 18 | 5 | NA |  | Yes | Yes | 5% | intranasal oxytocin | biomedical | 6 |  | static | % mouth-angry face | -0.04 |
| Le et al. (2022) | 18 | 5 | NA |  | Yes | Yes | 5% | intranasal oxytocin | biomedical | 6 |  | static | % mouth-fearful face | 0.19 |
| Le et al. (2022) | 18 | 5 | NA |  | Yes | Yes | 5% | intranasal oxytocin | biomedical | 6 |  | static | % mouth-happy face | 0.54 |
| Le et al. (2022) | 18 | 5 | NA |  | Yes | Yes | 5% | intranasal oxytocin | biomedical | 6 |  | static | % mouth-neutral face | -0.14 |
| Le et al. (2022) | 18 | 5 | NA |  | Yes | Yes | 5% | intranasal oxytocin | biomedical | 6 |  | dynamic | % total fixation duration for dynamic social | 0.12 |
| Le et al. (2022) | 18 | 5 | NA |  | Yes | Yes | 5% | intranasal oxytocin | biomedical | 6 |  | static | % eyes-angry face | 0.62 |
| Le et al. (2022) | 18 | 5 | NA |  | Yes | Yes | 5% | intranasal oxytocin | biomedical | 6 |  | static | % eyes-fearful face | -0.83 |
| Le et al. (2022) | 18 | 5 | NA |  | Yes | Yes | 5% | intranasal oxytocin | biomedical | 6 |  | static | % eyes-happy face | 0.38 |
| Le et al. (2022) | 18 | 5 | NA |  | Yes | Yes | 5% | intranasal oxytocin | biomedical | 6 |  | static | % eyes-neutral face | 0.17 |
| Le et al. (2022) | 18 | 5 | NA |  | Yes | Yes | 5% | intranasal oxytocin | biomedical | 6 |  | static | % nose-angry face | -0.30 |
| Le et al. (2022) | 18 | 5 | NA |  | Yes | Yes | 5% | intranasal oxytocin | biomedical | 6 |  | static | % nose-fearful face | -0.08 |
| Le et al. (2022) | 18 | 5 | NA |  | Yes | Yes | 5% | intranasal oxytocin | biomedical | 6 |  | static | % nose-happy face | -0.02 |
| Le et al. (2022) | 18 | 5 | NA |  | Yes | Yes | 5% | intranasal oxytocin | biomedical | 6 |  | static | % nose-neutral face | -0.13 |
| Le et al. (2022) | 18 | 5 | NA |  | Yes | Yes | 5% | intranasal oxytocin | biomedical | 6 |  | static | % mouth-angry face | 0.07 |
| Le et al. (2022) | 18 | 5 | NA |  | Yes | Yes | 5% | intranasal oxytocin | biomedical | 6 |  | static | % mouth-fearful face | -0.03 |
| Le et al. (2022) | 18 | 5 | NA |  | Yes | Yes | 5% | intranasal oxytocin | biomedical | 6 |  | static | % mouth-happy face | 0.18 |
| Le et al. (2022) | 18 | 5 | NA |  | Yes | Yes | 5% | intranasal oxytocin | biomedical | 6 |  | static | % mouth-neutral face | 0.19 |
| Le et al. (2022) | 5 | 5 | NA |  | Yes | Yes | 5% | intranasal oxytocin | biomedical | 6 |  | dynamic | % total fixation duration for dynamic social | 0.45 |
| Le et al. (2022) | 5 | 5 | NA |  | Yes | Yes | 5% | intranasal oxytocin | biomedical | 6 |  | static | % eyes-angry face | 0.07 |
| Le et al. (2022) | 5 | 5 | NA |  | Yes | Yes | 5% | intranasal oxytocin | biomedical | 6 |  | static | % eyes-fearful face | 0.68 |
| Le et al. (2022) | 5 | 5 | NA |  | Yes | Yes | 5% | intranasal oxytocin | biomedical | 6 |  | static | % eyes-happy face | -0.05 |
| Le et al. (2022) | 5 | 5 | NA |  | Yes | Yes | 5% | intranasal oxytocin | biomedical | 6 |  | static | % eyes-neutral face | -0.17 |
| Le et al. (2022) | 5 | 5 | NA |  | Yes | Yes | 5% | intranasal oxytocin | biomedical | 6 |  | static | % nose-angry face | 0.38 |
| Le et al. (2022) | 5 | 5 | NA |  | Yes | Yes | 5% | intranasal oxytocin | biomedical | 6 |  | static | % nose-fearful face | 0.23 |
| Le et al. (2022) | 5 | 5 | NA |  | Yes | Yes | 5% | intranasal oxytocin | biomedical | 6 |  | static | % nose-happy face | 0.27 |
| Le et al. (2022) | 5 | 5 | NA |  | Yes | Yes | 5% | intranasal oxytocin | biomedical | 6 |  | static | % nose-neutral face | 0.27 |
| Le et al. (2022) | 5 | 5 | NA |  | Yes | Yes | 5% | intranasal oxytocin | biomedical | 6 |  | static | % mouth-angry face | 0.50 |
| Le et al. (2022) | 5 | 5 | NA |  | Yes | Yes | 5% | intranasal oxytocin | biomedical | 6 |  | static | % mouth-fearful face | -0.24 |
| Le et al. (2022) | 5 | 5 | NA |  | Yes | Yes | 5% | intranasal oxytocin | biomedical | 6 |  | static | % mouth-happy face | 0.16 |
| Le et al. (2022) | 5 | 5 | NA |  | Yes | Yes | 5% | intranasal oxytocin | biomedical | 6 |  | static | % mouth-neutral face | -0.27 |
| Scherf et al. (2024) | 20 | 13.8 | 80 |  | Yes | No | 0% | Social Games for Autistic Adolescents (SAGA) | technology | 10 |  | static | total duration-task engagement | -0.01 |
| Scherf et al. (2024) | 20 | 13.8 | 80 |  | Yes | No | 0% | Social Games for Autistic Adolescents (SAGA) | technology | 10 |  | static | duration-face | -0.14 |
| Scherf et al. (2024) | 20 | 13.8 | 80 |  | Yes | No | 0% | Social Games for Autistic Adolescents (SAGA) | technology | 10 |  | static | duration-target object | 0.04 |
| Schmitt et al. (2023) | 15 | 20 | 100 |  | Yes | Yes | 0% | SB‑121 | biomedical | 4 |  | dynamic | social scene preference ratio | 0.57 |
| Tang et al. (2022) | 13 | 7.7 | 92 |  | Yes | Yes | 4% | storytelling with social contextual | behavioral | 4 |  | static | total fixation duration-photo | -0.73 |
| Tang et al. (2022) | 13 | 7.7 | 92 |  | Yes | Yes | 4% | storytelling with social contextual | behavioral | 4 |  | dynamic | total fixation duration-video | -0.07 |
| Tang et al. (2022) | 13 | 7.7 | 92 |  | Yes | Yes | 4% | storytelling with social contextual | behavioral | 4 |  | static | total visit duration-photo | -0.67 |
| Tang et al. (2022) | 13 | 7.7 | 92 |  | Yes | Yes | 4% | storytelling with social contextual | behavioral | 4 |  | dynamic | total visit duration-video | 0.08 |
| Tang et al. (2022) | 13 | 7.7 | 92 |  | Yes | Yes | 4% | storytelling with social contextual | behavioral | 4 |  | static | total fixation count-photo | -0.11 |
| Tang et al. (2022) | 13 | 7.7 | 92 |  | Yes | Yes | 4% | storytelling with social contextual | behavioral | 4 |  | dynamic | total fixation count-video | 0.16 |
| Tang et al. (2022) | 13 | 7.7 | 92 |  | Yes | Yes | 4% | storytelling without social contextual | behavioral | 4 |  | static | total fixation duration-photo | 0.20 |
| Tang et al. (2022) | 13 | 7.7 | 92 |  | Yes | Yes | 4% | storytelling without social contextual | behavioral | 4 |  | dynamic | total fixation duration-video | 0.03 |
| Tang et al. (2022) | 13 | 7.7 | 92 |  | Yes | Yes | 4% | storytelling without social contextual | behavioral | 4 |  | static | total visit duration-photo | 0.00 |
| Tang et al. (2022) | 13 | 7.7 | 92 |  | Yes | Yes | 4% | storytelling without social contextual | behavioral | 4 |  | dynamic | total visit duration-video | 0.17 |
| Tang et al. (2022) | 13 | 7.7 | 92 |  | Yes | Yes | 4% | storytelling without social contextual | behavioral | 4 |  | static | total fixation count-photo | 0.07 |
| Tang et al. (2022) | 13 | 7.7 | 92 |  | Yes | Yes | 4% | storytelling without social contextual | behavioral | 4 |  | dynamic | total fixation count-video | -0.15 |
| Tian et al. (2023) | 45 | 8.8 | 82 |  | No | No | 0% | transcranial magnetic stimulation | biomedical | 4 |  | static | fixation count | 0.36 |
| Tian et al. (2023) | 45 | 8.8 | 82 |  | No | No | 0% | transcranial magnetic stimulation | biomedical | 4 |  | static | fixation count | 0.58 |
| Tian et al. (2023) | 45 | 8.8 | 82 |  | No | No | 0% | transcranial magnetic stimulation | biomedical | 4 |  | static | fixation count | 0.29 |
| Tian et al. (2023) | 45 | 8.8 | 82 |  | No | No | 0% | transcranial magnetic stimulation | biomedical | 4 |  | static | total fixation duration | 0.46 |
| Tian et al. (2023) | 45 | 8.8 | 82 |  | No | No | 0% | transcranial magnetic stimulation | biomedical | 4 |  | static | total fixation duration | 0.79 |
| Tian et al. (2023) | 45 | 8.8 | 82 |  | No | No | 0% | transcranial magnetic stimulation | biomedical | 4 |  | static | total fixation duration | 0.39 |
| Umbricht et al. (2017) | 19 | 23.4 | 100 |  | Yes | Yes | 0% | RG7713 | biomedical | 2 |  | dynamic | activity monitoring ratio-activity | -0.29 |
| Umbricht et al. (2017) | 19 | 23.4 | 100 |  | Yes | Yes | 0% | RG7713 | biomedical | 2 |  | dynamic | activity monitoring ratio-background | 0.10 |
| Umbricht et al. (2017) | 19 | 23.4 | 100 |  | Yes | Yes | 0% | RG7713 | biomedical | 2 |  | dynamic | activity monitoring ratio-background with distractors | 0.10 |
| Umbricht et al. (2017) | 19 | 23.4 | 100 |  | Yes | Yes | 0% | RG7713 | biomedical | 2 |  | dynamic | activity monitoring ratio-body | -0.19 |
| Umbricht et al. (2017) | 19 | 23.4 | 100 |  | Yes | Yes | 0% | RG7713 | biomedical | 2 |  | dynamic | activity monitoring ratio-distractors | 0.10 |
| Umbricht et al. (2017) | 19 | 23.4 | 100 |  | Yes | Yes | 0% | RG7713 | biomedical | 2 |  | dynamic | activity monitoring ratio-head | 0.19 |
| Umbricht et al. (2017) | 19 | 23.4 | 100 |  | Yes | Yes | 0% | RG7713 | biomedical | 2 |  | dynamic | activity monitoring ratio-person | 0.10 |
| Umbricht et al. (2017) | 19 | 23.4 | 100 |  | Yes | Yes | 0% | RG7713 | biomedical | 2 |  | dynamic | biodetection-D-prime | -0.19 |
| Umbricht et al. (2017) | 19 | 23.4 | 100 |  | Yes | Yes | 0% | RG7713 | biomedical | 2 |  | dynamic | biomotion-latency | -0.38 |
| Umbricht et al. (2017) | 19 | 23.4 | 100 |  | Yes | Yes | 0% | RG7713 | biomedical | 2 |  | dynamic | biomotion-looking preference | -0.10 |
| Umbricht et al. (2017) | 19 | 23.4 | 100 |  | Yes | Yes | 0% | RG7713 | biomedical | 2 |  | dynamic | biomotion-orienting preference | 0.76 |
| Umbricht et al. (2017) | 19 | 23.4 | 100 |  | Yes | Yes | 0% | RG7713 | biomedical | 2 |  | dynamic | *Who’s Afraid of Virginia Woolf?*-background | -0.19 |
| Umbricht et al. (2017) | 19 | 23.4 | 100 |  | Yes | Yes | 0% | RG7713 | biomedical | 2 |  | dynamic | *Who’s Afraid of Virginia Woolf?*-body | 0.29 |
| Umbricht et al. (2017) | 19 | 23.4 | 100 |  | Yes | Yes | 0% | RG7713 | biomedical | 2 |  | dynamic | *Who’s Afraid of Virginia Woolf?*-eyes | 0.00 |
| Umbricht et al. (2017) | 19 | 23.4 | 100 |  | Yes | Yes | 0% | RG7713 | biomedical | 2 |  | dynamic | *Who’s Afraid of Virginia Woolf?*-head | 0.00 |
| Umbricht et al. (2017) | 19 | 23.4 | 100 |  | Yes | Yes | 0% | RG7713 | biomedical | 2 |  | dynamic | *Who’s Afraid of Virginia Woolf?*-mouth | -0.38 |
| Umbricht et al. (2017) | 19 | 23.4 | 100 |  | Yes | Yes | 0% | RG7713 | biomedical | 2 |  | static | gaze discrimination-eyes | 0.00 |
| Umbricht et al. (2017) | 19 | 23.4 | 100 |  | Yes | Yes | 0% | RG7713 | biomedical | 2 |  | static | gaze discrimination-inside face | -0.38 |
| Umbricht et al. (2017) | 19 | 23.4 | 100 |  | Yes | Yes | 0% | RG7713 | biomedical | 2 |  | static | gaze discrimination-mouth | 0.00 |
| Umbricht et al. (2017) | 19 | 23.4 | 100 |  | Yes | Yes | 0% | RG7713 | biomedical | 2 |  | static | gaze discrimination-nose | -0.29 |
| Umbricht et al. (2017) | 19 | 23.4 | 100 |  | Yes | Yes | 0% | RG7713 | biomedical | 2 |  | static | gender discrimination-eyes | -0.38 |
| Umbricht et al. (2017) | 19 | 23.4 | 100 |  | Yes | Yes | 0% | RG7713 | biomedical | 2 |  | static | gender discrimination-inside face | -0.48 |
| Umbricht et al. (2017) | 19 | 23.4 | 100 |  | Yes | Yes | 0% | RG7713 | biomedical | 2 |  | static | gender discrimination-mouth | 0.19 |
| Umbricht et al. (2017) | 19 | 23.4 | 100 |  | Yes | Yes | 0% | RG7713 | biomedical | 2 |  | static | gender discrimination-nose | -0.19 |
| Umbricht et al. (2017) | 19 | 23.4 | 100 |  | Yes | Yes | 0% | RG7713 | biomedical | 2 |  | dynamic | human activity-preference | 0.00 |
| Wieckowski & White (2020) | 8 | 10.9 | 63 |  | No | No | 13% | attention modification intervention | technology | 10 |  | dynamic | total duration of fixations on face | -0.88 |
| Wong, Tang, Koh et al. (2024) | 7 | NA | NA |  | No | No | 0% | oral folinic acid (leucovorin calcium) | biomedical | 12 |  | dynamic | total time spent watching video | -0.33 |
| Wong, Tang, Koh et al. (2024) | 7 | NA | NA |  | No | No | 0% | oral folinic acid (leucovorin calcium) | biomedical | 12 |  | dynamic | % time spent watching video | -0.33 |
| Wong, Tang, Koh et al. (2024) | 7 | NA | NA |  | No | No | 0% | oral folinic acid (leucovorin calcium) | biomedical | 12 |  | dynamic | % eyes | 0.38 |
| Wong, Tang, Koh et al. (2024) | 7 | NA | NA |  | No | No | 0% | oral folinic acid (leucovorin calcium) | biomedical | 12 |  | dynamic | % mouth | 0.02 |
| Wong, Tang, Koh et al. (2024) | 7 | NA | NA |  | No | No | 0% | oral folinic acid (leucovorin calcium) | biomedical | 12 |  | dynamic | % body | -0.21 |
| Wong, Tang, Koh et al. (2024) | 7 | NA | NA |  | No | No | 0% | oral folinic acid (leucovorin calcium) | biomedical | 12 |  | dynamic | % hands | 0.00 |
| Wong, Tang, Koh et al. (2024) | 7 | NA | NA |  | No | No | 0% | oral folinic acid (leucovorin calcium) | biomedical | 24 |  | dynamic | total time spent watching video | -0.26 |
| Wong, Tang, Koh et al. (2024) | 7 | NA | NA |  | No | No | 0% | oral folinic acid (leucovorin calcium) | biomedical | 24 |  | dynamic | % time spent watching video | -0.27 |
| Wong, Tang, Koh et al. (2024) | 7 | NA | NA |  | No | No | 0% | oral folinic acid (leucovorin calcium) | biomedical | 24 |  | dynamic | % eyes | 0.50 |
| Wong, Tang, Koh et al. (2024) | 7 | NA | NA |  | No | No | 0% | oral folinic acid (leucovorin calcium) | biomedical | 24 |  | dynamic | % mouth | -0.19 |
| Wong, Tang, Koh et al. (2024) | 7 | NA | NA |  | No | No | 0% | oral folinic acid (leucovorin calcium) | biomedical | 24 |  | dynamic | % body | -0.98 |
| Wong, Tang, Koh et al. (2024) | 7 | NA | NA |  | No | No | 0% | oral folinic acid (leucovorin calcium) | biomedical | 24 |  | dynamic | % hands | 0.19 |
| Wong, Tang, Riard et al. (2024) | 13 | NA | NA |  | No | No | 0% | autologous umbilical cord blood | biomedical | 26.07 |  | dynamic | total time spent watching video | 0.07 |
| Wong, Tang, Riard et al. (2024) | 13 | NA | NA |  | No | No | 0% | autologous umbilical cord blood | biomedical | 26.07 |  | dynamic | % time spent watching video | 0.07 |
| Wong, Tang, Riard et al. (2024) | 13 | NA | NA |  | No | No | 0% | autologous umbilical cord blood | biomedical | 26.07 |  | dynamic | % total time spent in correct AOI | 0.66 |
| Wong, Tang, Riard et al. (2024) | 13 | NA | NA |  | No | No | 0% | autologous umbilical cord blood | biomedical | 26.07 |  | dynamic | % eyes | 0.42 |
| Wong, Tang, Riard et al. (2024) | 13 | NA | NA |  | No | No | 0% | autologous umbilical cord blood | biomedical | 26.07 |  | dynamic | % mouth | 0.03 |
| Wong, Tang, Riard et al. (2024) | 13 | NA | NA |  | No | No | 0% | autologous umbilical cord blood | biomedical | 26.07 |  | dynamic | % body | -0.15 |
| Wong, Tang, Riard et al. (2024) | 13 | NA | NA |  | No | No | 0% | autologous umbilical cord blood | biomedical | 26.07 |  | dynamic | % hands | 0.07 |
| Wong, Tang, Riard et al. (2024) | 19 | 4.15 | 79 |  | No | No | 0% | autologous umbilical cord blood | biomedical | 52.14 |  | dynamic | total time spent watching video | -0.01 |
| Wong, Tang, Riard et al. (2024) | 19 | 4.15 | 79 |  | No | No | 0% | autologous umbilical cord blood | biomedical | 52.14 |  | dynamic | % time spent watching video | -0.02 |
| Wong, Tang, Riard et al. (2024) | 19 | 4.15 | 79 |  | No | No | 0% | autologous umbilical cord blood | biomedical | 52.14 |  | dynamic | % total time spent in correct AOI | 0.18 |
| Wong, Tang, Riard et al. (2024) | 19 | 4.15 | 79 |  | No | No | 0% | autologous umbilical cord blood | biomedical | 52.14 |  | dynamic | % eyes | 0.07 |
| Wong, Tang, Riard et al. (2024) | 19 | 4.15 | 79 |  | No | No | 0% | autologous umbilical cord blood | biomedical | 52.14 |  | dynamic | % mouth | 0.02 |
| Wong, Tang, Riard et al. (2024) | 19 | 4.15 | 79 |  | No | No | 0% | autologous umbilical cord blood | biomedical | 52.14 |  | dynamic | % body | -0.17 |
| Wong, Tang, Riard et al. (2024) | 19 | 4.15 | 79 |  | No | No | 0% | autologous umbilical cord blood | biomedical | 52.14 |  | dynamic | % hands | 0.45 |
| Yamasue et al. (2020) | 78 | 27.6 | 100 |  | Yes | Yes | 3% | intranasal oxytocin | biomedical | 6 |  | dynamic | % fixation time on eyes (still image) | 0.08 |
| Yamasue et al. (2020) | 78 | 27.6 | 100 |  | Yes | Yes | 3% | intranasal oxytocin | biomedical | 6 |  | dynamic | % fixation time on eyes (blinking) | 0.11 |
| Yamasue et al. (2020) | 78 | 27.6 | 100 |  | Yes | Yes | 3% | intranasal oxytocin | biomedical | 6 |  | dynamic | % fixation time on eyes (mouse moving) | 0.09 |
| Yamasue et al. (2020) | 78 | 27.6 | 100 |  | Yes | Yes | 3% | intranasal oxytocin | biomedical | 6 |  | dynamic | % fixation time on eyes (silent) | 0.19 |
| Yamasue et al. (2020) | 78 | 27.6 | 100 |  | Yes | Yes | 3% | intranasal oxytocin | biomedical | 6 |  | dynamic | % fixation time on eyes (talking) | 0.54 |
| Yamasue et al. (2020) | 78 | 27.6 | 100 |  | Yes | Yes | 3% | intranasal oxytocin | biomedical | 6 |  | dynamic | % fixation time on upright | 0.32 |
| Yamasue et al. (2020) | 78 | 27.6 | 100 |  | Yes | Yes | 3% | intranasal oxytocin | biomedical | 6 |  | dynamic | % fixation time on people | 0.28 |
| Yamasue et al. (2020) | 78 | 27.6 | 100 |  | Yes | Yes | 3% | intranasal oxytocin | biomedical | 6 |  | dynamic | % fixation time on geometry | 0.33 |
| Yamasue et al. (2020) | 78 | 27.6 | 100 |  | Yes | Yes | 3% | intranasal oxytocin | biomedical | 6 |  | dynamic | % fixation time on pointed (social region) | 0.12 |
| Zamzow et al. (2014) | 12 | 18.25 | 75 |  | Yes | Yes | 0% | propranolol | biomedical | 0.29 |  | dynamic | absolute mean fixation time on mouth | 0.54 |
| Zamzow et al. (2014) | 12 | 18.25 | 75 |  | Yes | Yes | 0% | propranolol | biomedical | 0.29 |  | dynamic | absolute mean fixation time on eye | 0.13 |
| Zamzow et al. (2014) | 12 | 18.25 | 75 |  | Yes | Yes | 0% | propranolol | biomedical | 0.29 |  | dynamic | proportion of total fixation time on mouth | 0.50 |
| Zamzow et al. (2014) | 12 | 18.25 | 75 |  | Yes | Yes | 0% | propranolol | biomedical | 0.29 |  | dynamic | proportion of total fixation time on eye | 0.19 |
| Zhao et al. (2024) | 26 | NA | 96 |  | Yes | Yes | 0% | intranasal  oxytocin | biomedical | 6 |  | dynamic | % total time spent viewing the dynamic social stimuli relative to the dynamic geometric stimuli | 0.35 |
| Zhao et al. (2024) | 26 | NA | 96 |  | Yes | Yes | 0% | intranasal oxytocin | biomedical | 6 |  | static | % eyes-angry face | 0.61 |
| Zhao et al. (2024) | 26 | NA | 96 |  | Yes | Yes | 0% | intranasal oxytocin | biomedical | 6 |  | static | % nose-neutral face | 0.56 |

*Note*. Reported treatment durations (days, weeks, months) are converted to weeks for consistency in comparison. For Wong, Tang, Riard et al. (2024), the treatment duration refers to the period from a one-time treatment to follow-up. NA = data not available in the study. ABA = applied behavior analysis. EI = early intervention. IJA = initiating joint attention. JA = joint attention. RJA = responding to joint attention.

**SUPPLEMENTARY TABLE 5** Characteristics of included studies with effect sizes associated with correlation between baseline (i.e., pre-treatment) eye-tracking outcomes and changes in developmental outcomes from pre- to post-treatment (*k* = 39 effect sizes).

| **Authors (year)** | **Autistic Participants** | | |  | **Treatment** | | | | |  | **Clinical Outcome** | |  | **Eye Tracking** | | ***z*** |
| --- | --- | --- | --- | --- | --- | --- | --- | --- | --- | --- | --- | --- | --- | --- | --- | --- |
|  | ***N*** | **mean age (years)** | **sex (%male)** |  | **random ization** | **blinding** | **drop out (%)** | **name** | **duration**  **(weeks)** |  | **domain** | **measure** |  | **stimulus motion format** | **measure** |  |
| Bent et al. (2023) | 82 | 3.1 | 79 |  | No | No | 0% | ESDM or EIBI | 52.1 |  | cognitive | NVDQ |  | dynamic | total duration of gaze to animation | 0.35 |
| Bent et al. (2023) | 82 | 3.1 | 79 |  | No | No | 0% | ESDM or EIBI | 52.1 |  | cognitive | NVDQ |  | both | average duration of attention to social and non-social targets | 0.14 |
| Bent et al. (2023) | 82 | 3.1 | 79 |  | No | No | 0% | ESDM or EIBI | 52.1 |  | cognitive | NVDQ |  | dynamic | total duration of attention to target | 0.27 |
| Bent et al. (2023) | 82 | 3.1 | 79 |  | No | No | 0% | ESDM or EIBI | 52.1 |  | cognitive | NVDQ |  | dynamic | proportion of trials with first look to target | 0.27 |
| Bent et al. (2023) | 82 | 3.1 | 79 |  | No | No | 0% | ESDM or EIBI | 52.1 |  | cognitive | NVDQ |  | dynamic | total duration of attention to playful actor | 0.35 |
| Bent et al. (2023) | 82 | 3.1 | 79 |  | No | No | 0% | ESDM or EIBI | 52.1 |  | cognitive | VDQ |  | dynamic | total duration of gaze to animation | 0.33 |
| Bent et al. (2023) | 82 | 3.1 | 79 |  | No | No | 0% | ESDM or EIBI | 52.1 |  | cognitive | VDQ |  | both | average duration of attention to social and non-social targets | 0.27 |
| Bent et al. (2023) | 82 | 3.1 | 79 |  | No | No | 0% | ESDM or EIBI | 52.1 |  | cognitive | VDQ |  | dynamic | total duration of attention to target | 0.37 |
| Bent et al. (2023) | 82 | 3.1 | 79 |  | No | No | 0% | ESDM or EIBI | 52.1 |  | cognitive | VDQ |  | dynamic | proportion of trials with first look to target | 0.20 |
| Bent et al. (2023) | 82 | 3.1 | 79 |  | No | No | 0% | ESDM or EIBI | 52.1 |  | cognitive | VDQ |  | dynamic | total duration of attention to playful actor | 0.56 |
| Bent et al. (2023) | 82 | 3.1 | 79 |  | No | No | 0% | ESDM or EIBI | 52.1 |  | behavioral | VABS-ABC |  | dynamic | total duration of gaze to animation | 0.20 |
| Bent et al. (2023) | 82 | 3.1 | 79 |  | No | No | 0% | ESDM or EIBI | 52.1 |  | behavioral | VABS-ABC |  | both | average duration of attention to social and non-social targets | 0.17 |
| Bent et al. (2023) | 82 | 3.1 | 79 |  | No | No | 0% | ESDM or EIBI | 52.1 |  | behavioral | VABS-ABC |  | dynamic | total duration of attention to target | 0.20 |
| Bent et al. (2023) | 82 | 3.1 | 79 |  | No | No | 0% | ESDM or EIBI | 52.1 |  | behavioral | VABS-ABC |  | dynamic | proportion of trials with first look to target | 0.13 |
| Bent et al. (2023) | 82 | 3.1 | 79 |  | No | No | 0% | ESDM or EIBI | 52.1 |  | behavioral | VABS-ABC |  | dynamic | total duration of attention to playful actor | 0.37 |
| Green et al. (2022) | 20 | 17.6 | 75 |  | No | No | 0% | social skills intervention | 8 |  | behavioral | SRS-SA |  | dynamic | proportion of total fixation duration on face | 0.50 |
| Green et al. (2022) | 20 | 17.6 | 75 |  | No | No | 0% | social skills intervention | 8 |  | behavioral | SRS-SC |  | dynamic | proportion of total fixation duration on face | 0.46 |
| Green et al. (2022) | 20 | 17.6 | 75 |  | No | No | 0% | social skills intervention | 8 |  | behavioral | SRS-SC |  | dynamic | proportion of total fixation duration on background | 0.76 |
| Green et al. (2022) | 20 | 17.6 | 75 |  | No | No | 0% | social skills intervention | 8 |  | behavioral | SRS-RRB |  | dynamic | proportion of total fixation duration on background | 0.62 |
| Green et al. (2022) | 20 | 17.6 | 75 |  | No | No | 0% | social skills intervention | 8 |  | behavioral | SRS-total |  | dynamic | proportion of total fixation duration on background | 0.56 |
| Green et al. (2022) | 20 | 17.6 | 75 |  | No | No | 0% | social skills intervention | 8 |  | behavioral | SRS-SC |  | dynamic | proportion of total fixation duration on hands/objects | -0.93 |
| Green et al. (2022) | 20 | 17.6 | 75 |  | No | No | 0% | social skills intervention | 8 |  | behavioral | SRS-RRB |  | dynamic | proportion of total fixation duration on hands/objects | -0.73 |
| Green et al. (2022) | 20 | 17.6 | 75 |  | No | No | 0% | social skills intervention | 8 |  | behavioral | SRS-total |  | dynamic | proportion of total fixation duration on hands/objects | -0.89 |
| Robain et al. (2020) | 60 | 3 | 100 |  | No | No | 0% | ESDM or community treatment | 52.1 |  | behavioral | ADOS-RRB |  | dynamic | % social orienting | -0.13 |
| Robain et al. (2020) | 60 | 3 | 100 |  | No | No | 0% | ESDM or community treatment | 52.1 |  | behavioral | ADOS-SA |  | dynamic | % social orienting | -0.11 |
| Robain et al. (2020) | 60 | 3 | 100 |  | No | No | 0% | ESDM or community treatment | 52.1 |  | behavioral | ADOS-total |  | dynamic | % social orienting | -0.12 |
| Robain et al. (2020) | 60 | 3 | 100 |  | No | No | 0% | ESDM or community treatment | 52.1 |  | cognitive | DQ |  | dynamic | % social orienting | 0.11 |
| Vivanti et al. (2013) | 21 | 3.2 | 95 |  | No | No | 0% | ESDM | 52.1 |  | cognitive | MSEL-VR |  | dynamic | amount of attention to actor's face vs. objects | 0.10 |
| Vivanti et al. (2013) | 21 | 3.2 | 95 |  | No | No | 0% | ESDM | 52.1 |  | cognitive | MSEL-FM |  | dynamic | amount of attention to actor's face vs. objects | -0.17 |
| Vivanti et al. (2013) | 21 | 3.2 | 95 |  | No | No | 0% | ESDM | 52.1 |  | cognitive | MSEL-RL |  | dynamic | amount of attention to actor's face vs. objects | 0.15 |
| Vivanti et al. (2013) | 21 | 3.2 | 95 |  | No | No | 0% | ESDM | 52.1 |  | cognitive | MSEL-EL |  | dynamic | amount of attention to actor's face vs. objects | 0.23 |
| Vivanti et al. (2013) | 21 | 3.2 | 95 |  | No | No | 0% | ESDM | 52.1 |  | cognitive | MSEL-VR |  | dynamic | number of fixations to target vs. other objects | 0.30 |
| Vivanti et al. (2013) | 21 | 3.2 | 95 |  | No | No | 0% | ESDM | 52.1 |  | cognitive | MSEL-FM |  | dynamic | number of fixations to target vs. other objects | 0.09 |
| Vivanti et al. (2013) | 21 | 3.2 | 95 |  | No | No | 0% | ESDM | 52.1 |  | cognitive | MSEL-RL |  | dynamic | number of fixations to target vs. other objects | 0.65 |
| Vivanti et al. (2013) | 21 | 3.2 | 95 |  | No | No | 0% | ESDM | 52.1 |  | cognitive | MSEL-EL |  | dynamic | number of fixations to target vs. other objects | 0.23 |
| Vivanti et al. (2013) | 21 | 3.2 | 95 |  | No | No | 0% | ESDM | 52.1 |  | behavioral | ADOS-SA |  | dynamic | amount of attention to actor's face vs. objects | 0.01 |
| Vivanti et al. (2013) | 21 | 3.2 | 95 |  | No | No | 0% | ESDM | 52.1 |  | behavioral | ADOS-RBB |  | dynamic | amount of attention to actor's face vs. objects | 0.12 |
| Vivanti et al. (2013) | 21 | 3.2 | 95 |  | No | No | 0% | ESDM | 52.1 |  | behavioral | ADOS-SA |  | dynamic | number of fixations to target vs. other objects | -0.35 |
| Vivanti et al. (2013) | 21 | 3.2 | 95 |  | No | No | 0% | ESDM | 52.1 |  | behavioral | ADOS-RBB |  | dynamic | number of fixations to target vs. other objects | -0.24 |

*Note*. All interventions in the included studies were behavioral. Reported treatment durations (weeks, year) are converted to weeks for consistency in comparison ADOS-RRB = Autism Diagnostic Observation Schedule-restricted repetitive behavior; ADOS-SA = ADOS-social affect; DQ = Developmental Quotient; EIBI = Early Intensive Behavioural Intervention; ESDM = Early Start Denver Model; MSEL-EL = Mullen Scales of Early Learning-expressive language; MSEL-FM = MSEL-fine motor; MSEL-RL = MSEL-receptive language; MSEL-VR = MSEL-visual reception; NVDQ = nonverbal DQ; SRS-RRB = Social Responsiveness Scale-restricted interests and repetitive behaviors; SRS-SA = SRS-social awareness; SRS-SC = SRS-social communication; VDQ = verbal DQ; VABS-ABC = Vineland Adaptive Behavior Scale-adaptive behavior composite.
